# Serious adverse events and 30-day and 90-day re-admission rates following anatomic versus reverse total shoulder arthroplasty: a systematic review and meta-analysis

**DOI:** 10.1016/j.xrrt.2026.100875

**Published:** 2026-09-04

**Authors:** Hussayn Shinwari, Usman Tahir, Ahmed Popal, Hanan Taimur Shinwari, Deniz Vural, Wisal Khan, Arnau Sheikh, Jawwad Mihran Haider, Abith Ganesh Kamath, Saran Singh Gill, Kapil Sugand

**Affiliations:** a Worcestershire Acute Hospitals NHS Trust, Worcestershire Royal Hospital, Worcester, UK; b Barts Health NHS Trust, The Royal London Hospital, London, UK; c St George's University Hospitals NHS Foundation Trust, St George's Hospital, London, UK; d Faculty of Medicine, University of Sheffield, Sheffield, UK; e City St George’s, University of London, London, UK; f Institute of Biotechnology and Genetic Engineering (IBGE), The University of Agriculture, Peshawar, Pakistan; g Faculty of Medicine, Imperial College London, London, UK

**Keywords:** Reverse total shoulder arthroplasty, Anatomic total shoulder arthroplasty, Early complications, Serious adverse events, 30-day re-admission, 90-day re-admission

## Abstract

**Background:**

Reverse total shoulder arthroplasty (rTSA) has expanded rapidly, but its early post-operative outcomes relative to anatomic total shoulder arthroplasty (aTSA) remain unclear. This systematic review and meta-analysis evaluate serious adverse events (SAEs) and 30-day and 90-day re-admission rates following primary aTSA and rTSA.

**Methods:**

A systematic search of PubMed, Embase, Medline, Scopus, and Web of Science was performed through July 2025. Studies reporting SAEs or early re-admissions after primary total shoulder arthroplasty were included. Risk of bias was assessed using Risk of bias was assessed using the Risk Of Bias In Non-randomized Studies of Interventions (ROBINS-I) tool and the Cochrane Risk of Bias 2 (RoB 2) tool. Random-effects meta-analyses were conducted to estimate pooled event rates and comparative odds ratios (ORs) between rTSA and aTSA, and single-arm meta-analyses to determine pooled event rates for each procedure.

**Results:**

Forty-six studies encompassing 1,445,486 patients (rTSA: 885,825; aTSA: 559,661) were included. Comparative analyses demonstrated that rTSA was associated with higher odds of SAEs (OR: 1.38; 95% confidence interval [CI]: 1.10-1.74), 30-day re-admission (OR: 1.71; 95% CI: 1.47-1.98), and 90-day re-admission (OR: 1.53; 95% CI: 1.34-1.75) compared with aTSA. Single-arm pooled rates confirmed higher absolute event rates for rTSA (SAE 8.78% vs. 5.28%; 30-day re-admission 3.16% vs. 1.24%; 90-day re-admission 4.09% vs. 3.05%). Both procedures exhibited low overall mortality, but rTSA had increased dislocation, infection, and revision rates, whereas aTSA showed higher mechanical loosening and rotator cuff failure.

**Conclusion:**

rTSA carries higher perioperative morbidity and early re-admission compared with aTSA, although absolute rates remain low. These findings underscore the importance of procedure-specific patient selection, comorbidity optimization, and tailored perioperative care to mitigate early complications and enhance value-based shoulder arthroplasty outcomes.

Total shoulder arthroplasty (TSA) is widely used as a definitive surgical treatment for degenerative and traumatic disorders of the shoulder complex, including advanced glenohumeral osteoarthritis, significant rotator cuff pathology, and complex proximal humerus fractures affecting joint function.^45^ In the United States, reverse total shoulder arthroplasty (rTSA) accounted for 55% of cases in 2016 and nearly 70% by 2020, with annual volumes increasing from 14,781 to 23,644 procedures.^38^ Overall TSA volume increased by 104% between 2011 and 2017, and projections suggest cumulative anatomic total shoulder arthroplasty (aTSA) in the United States will exceed 10 million by 2060, with annual growth of 11.7%.^11^^,^^57^ These trends reflect a global shift in surgical practice, expanding indications, and an aging population, with rTSA now dominating contemporary shoulder arthroplasty practice.

As TSA volumes rise, early post-operative outcomes have become a critical focus, with 30-day re-admissions reflecting perioperative safety and 90-day re-admissions capturing broader early complications. Serious adverse events (SAEs) including infection, dislocation, and thromboembolic events account for up to 63% of 90-day re-admissions and substantially increase morbidity.^8^^,^^48^ Early complications also impose significant economic burden with 90-day re-admissions and re-operations exceeding $100,000 per patient, a 2.6-fold increase compared with uncomplicated cases.^7^^,^^48^ These data highlight the importance of procedure-specific risk stratification to guide clinical decision-making, optimize perioperative planning, and support value-based care initiatives.

To date, no systematic review has evaluated SAEs alongside 30-day and 90-day re-admissions following TSA with stratification by procedure type. With the rapid rise of rTSA and the increasing use of early post-operative outcomes as quality metrics, this review and meta-analysis aim to provide robust, procedure-specific estimates, establishing benchmarks to guide clinical decision-making, patient counseling, and healthcare policy.

## Materials and methods

### Adherence to guidelines

The systematic review was conducted in line with the Preferred Reporting Items for Systematic Reviews and Meta-Analyses (PRISMA) 2020 guidelines.^43^ The completed PRISMA flowchart was shown in Figure 1.

**Figure 1:**
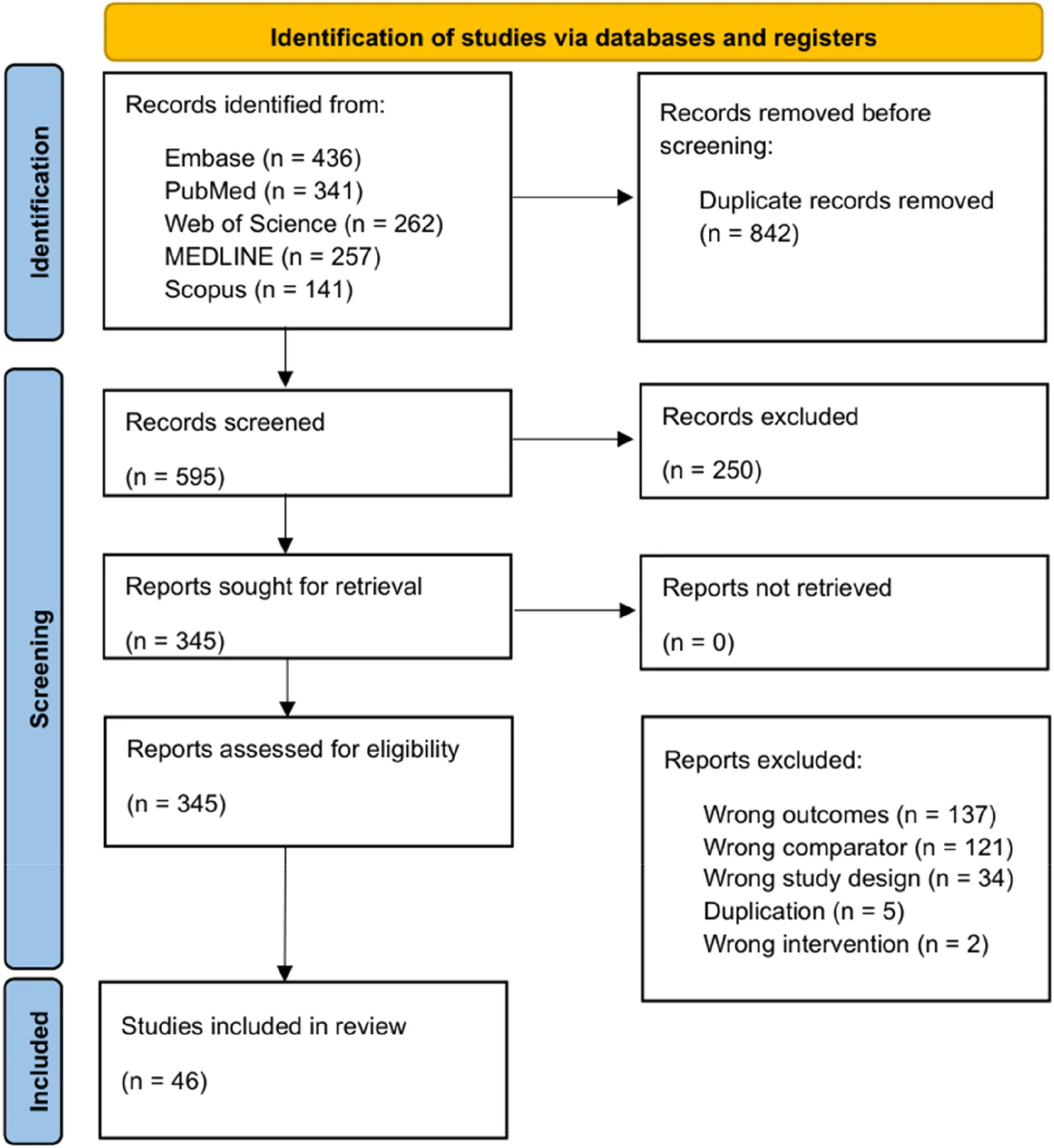
PRISMA flow diagram. *PRISMA*, Preferred Reporting Items for Systematic Reviews and Meta-Analyses.

PROSPERO registration: CRD420261328949.

### Electronic databases

A multidatabase search was performed from conception to July 20, 2025, encompassing PubMed, Embase, Medline, Scopus, and Web of Science. Our full search string can be found in Supplementary Table S1.

### Eligibility criteria

Eligible studies included adult patients (aged ≥18 years) undergoing primary anatomic or primary reverse TSA, that reported SAEs and/or 30-day or 90-day re-admission rates in the early post-operative period. Both comparative studies evaluating outcomes between anatomic and reverse TSA and single-arm studies reporting procedure-specific outcomes were included. Eligible study designs were observational studies, including retrospective or prospective cohort studies and registry-based or database-based analyses. Studies were excluded if they involved pediatric populations, revision or non-arthroplasty shoulder procedures, no clear surgical approach, or did not report extractable early post-operative adverse event or re-admission data. Only full-text articles published in English were considered. Supplementary Table S2 outlines the Population, Intervention, Comparison, and Outcomes criteria.

### Screening and data extraction

Studies were imported into Covidence software, where duplicates were removed. Three reviewers (H.S., U.T., and A.P.) initially screened titles and abstracts, following Cochrane Handbook recommendations to retain potentially relevant studies. Full-text articles were reviewed for eligibility based on the Population, Intervention, Comparison, and Outcomes framework. Data extraction was performed independently by 5 reviewers (H.S., U.T., A.P., H.T.S., and D.V.). Discrepancies were resolved by discussion, with final arbitration by the senior author (K.S.). Data were extracted using an Excel (Microsoft Inc., Washington, USA) spreadsheet including study characteristics, study design, patient demographics, location, publication year, sample size, and outcomes assessed.

### Risk of bias

Four independent reviewers (U.T., A.P., H.T.S., and D.V.) assessed the risk of bias using ROBINS-I for non-randomized studies and RoB 2 for randomized control trials as shown in Figures 2 and 3.^50^^,^^51^ Disagreements were resolved through discussion, with a fifth reviewer (H.S.) providing arbitration when necessary. The risk of bias assessment focused on domains like randomization, blinding, and handling of missing data to ensure result validity.

**Figure 2:**
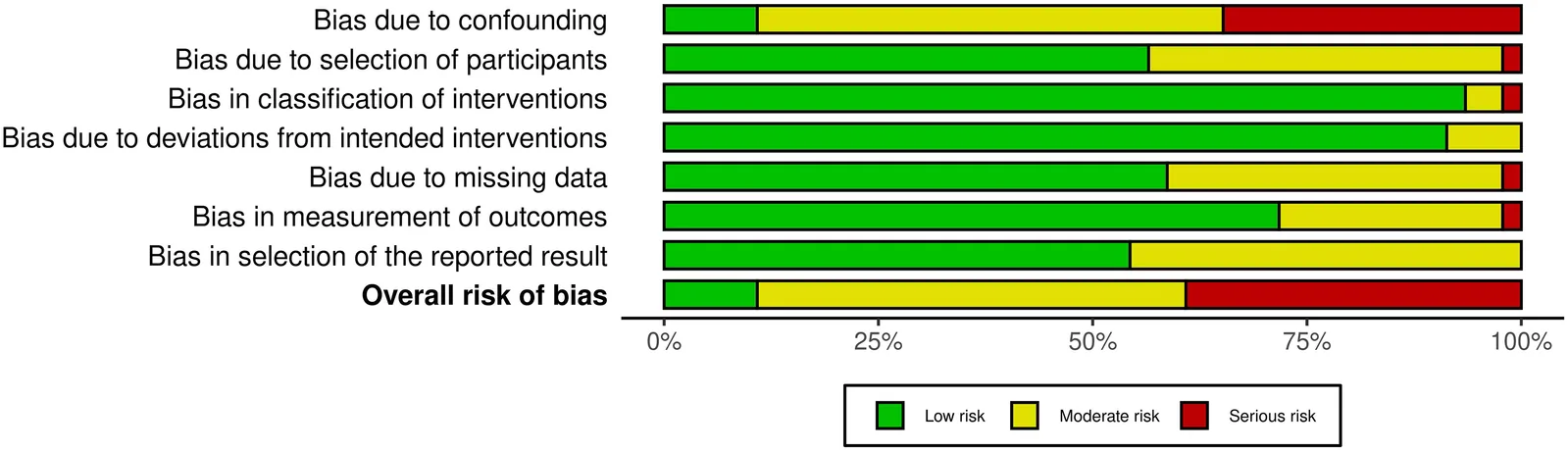
ROBIN-I risk of bias. *ROBINS-I,* Risk Of Bias In Non-randomized Studies of Interventions.

**Figure 3:**
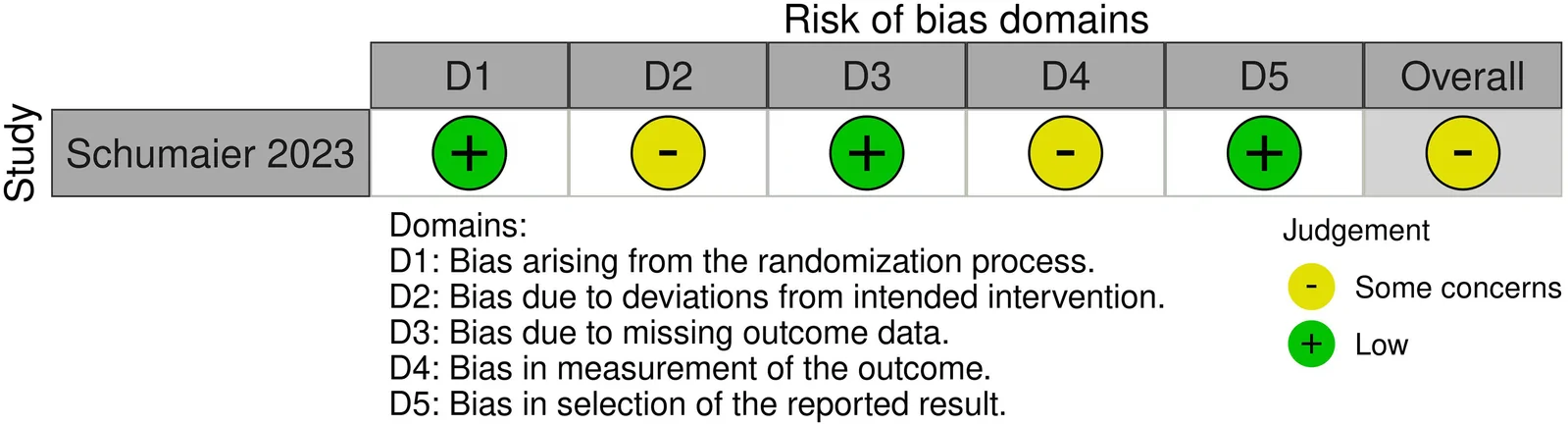
RoB-2 risk of bias.

### Statistical analysis

All statistical analyses were performed using RStudio (version 2024.04.2 + 764; R Foundation for Statistical Computing) using the meta package, in accordance with PRISMA guidelines.

### Comparative meta-analysis

Between-group comparisons (aTSA vs. rTSA) were assessed using comparative meta-analysis with odds ratios (ORs) as the effect measure, with 95% confidence intervals (CIs) reported. Only studies reporting data for both comparison arms were included. Random-effects models were used, with between-study variance estimated using restricted maximum likelihood. A continuity correction of 0.5 was applied across all studies. Comparative meta-analysis was performed when at least 3 studies were available for a given comparison. Data were presented as proportions and ORs.

### Single-arm meta-analysis

For each outcome (SAE, 30-day events, and 90-day events), single-arm meta-analyses were conducted separately for aTSA and rTSA. Meta-analysis was undertaken when at least 3 studies reported valid data for a given outcome and treatment group. Pooled event proportions were estimated using random-effects models with a logit transformation of proportions, and between-study variance (τ^2^) was estimated using maximum likelihood. A continuity correction of 0.5 was applied uniformly to accommodate zero-event studies. Results are presented as pooled proportions (%) with 95% CIs.

Because single-arm meta-analysis does not involve a comparator, no *P* value for effect was reported for pooled proportions. Accordingly, statistical inference for single-arm analyses focused on the magnitude of the pooled proportion, its CI, and the degree of between-study heterogeneity.

### Assessment of heterogeneity and publication bias

Between-study heterogeneity was assessed using Cochran's Q test, the I^2^ statistic (expressed as a percentage), and the between-study variance (τ^2^). The I^2^ statistic represents the proportion of total variability attributable to between-study heterogeneity rather than sampling error. Publication bias was evaluated only for comparative meta-analyses using contour-enhanced funnel plots, with shading corresponding to conventional levels of statistical significance (*P* < .10, *P* < .05, and *P* < .01). All statistical tests were 2-sided, and *P* < .05 was considered statistically significant.

## Results

### Study selection

A total of 1,437 articles were initially identified (Fig. 1). After removal of duplicates and detailed screening against predefined inclusion and exclusion criteria, 46 studies involving a total of 1,445,486 (rTSA: 885,825, aTSA: 559,661) patients were deemed eligible and included in the final analysis.^2^, ^3^, ^4^, ^5^, ^6^^,^^8^, ^9^, ^10^^,^^12^, ^13^, ^14^, ^15^, ^16^, ^17^, ^18^^,^^20^, ^21^, ^22^, ^23^, ^24^, ^25^, ^26^^,^^28^, ^29^, ^30^, ^31^, ^32^, ^33^, ^34^, ^35^, ^36^, ^37^^,^^39^, ^40^, ^41^, ^42^^,^^44^^,^^46^^,^^47^^,^^49^^,^^52^, ^53^, ^54^, ^55^, ^56^^,^^58^

### Study characteristics

The mean (or median) age of patients across studies ranged from 18 to more than 90 years, with most cohorts reporting a mean between 55 and 76 years. Gender distribution varied widely across studies. Mean body mass index, where reported, ranged from 26.0 to 42.4 kg/m^2^. Sample sizes differed substantially, from as few as 76 patients in small single-center cohorts to more than 428,000 patients in large national database studies (Tables I and II).

**Table I:** Study characteristics.

Author (yr)	Title	Country/setting	Study design	Journal	Sample size	Age (mean/median, SD or IQR)	BMI (mean/median, SD or IQR)	% male	Follow-up duration
Mastrokostas,^36^ 2025	Outcomes of ambulatory vs. outpatient hospital-based surgical center shoulder arthroplasty: complications, readmissions, and charges	United States	Retrospective cohort	European Journal of Orthopaedic Surgery & Traumatology	56,160 total (ASCs: 9,367; HSCs: 46,793) ambulatory surgical centers (ASCs) & hospital-based surgical centers (HSCs)			45.60%	Medical complications: 90 dImplant-related complications: 2 yrs
Mayfield,^37^ 2025	Effect of body mass index on complications following total shoulder arthroplasty: a population-wide analysis	United States	Retrospective cohort	Journal of Shoulder and Elbow Surgery	Total: 32,645 patientsaTSA: 10,951rTSA: 21,694	aTSA BMI <30: 67.97 ± 9.58 yrs; BMI 30–35: 66.89 ± 8.99 yrs; BMI >35: 64.87 ± 8.97 yrsrTSA BMI <30: 72.71 ± 8.63 yrs; BMI 30–35: 71.11 ± 8.12 yrs; BMI >35: 69.01 ± 7.87 yrs	BMI reported only as categorical cohorts (<30, 30–35, >35 kg/m^2^)		90 d
Marigi,^35^ 2023	Higher rates of mortality and perioperative complications in patients undergoing primary shoulder arthroplasty and a history of previous stroke	United States	Retrospective cohort	Journal of Shoulder and Elbow Surgery	• Stroke cohort: 205• Matched cohort: 410	• Stroke cohort: 72.4 ± 9.6 yrs• Matched cohort: 72.3 ± 9.5 yrs• Age at first stroke: 63.7 ± 13.6 yrs	• Stroke cohort: 31.1 ± 7.4• Matched cohort: 30.4 ± 6.0	• Stroke cohort: 42.4% (87/205)• Matched cohort: 42.4% (174/410)	• Stroke cohort: 6.6 ± 4.5 yrs• Matched cohort: 6.1 ± 4.1 yrs
Seetharam,^49^ 2022	Trends in outpatient shoulder arthroplasty during the COVID-19 (coronavirus disease 2019) era: increased proportion of outpatient cases with decrease in 90-day readmissions	United States	Retrospective cohort	J Shoulder Elbow Surg	Total: 567 patients (after exclusions from 736 screened; 169 excluded).Pre–COVID-19 era: 297 patients.COVID-19 era: 270 patients.Minimum 90-d follow-up rate: 93% (526/567).	Pre–COVID-19: 68.6 ± 8.8 yrsCOVID-19 era: 68.3 ± 8.9 yrsCOVID-era sub-group:Inpatient: 70.6 ± 8.1 yrsOutpatient: 64.1 ± 7.6 yrs.	Pre–COVID-19: 32.3 ± 7.2.COVID-19 era: 31.7 ± 7.3.COVID-era sub-group:Inpatient: 32.1 ± 7.4.Outpatient: 31.1 ± 7.7.	Pre–COVID-19: 123/297 = 41.4% male.COVID-19 era: 126/270 = 46.7% male.COVID-era sub-group (reported in table):Inpatient: 86 males (table lists 53.2%) — raw n = 86/184.Outpatient: 47 males (table lists 54.7%) — raw n = 47/86.	Minimum 90 d for outcomes (90-d outcome window)
Antonacci,^5^ 2021	Complications and Readmissions after Reverse and Anatomic Total Shoulder Arthroplasty with Same-day Discharge	United States	Retrospective cohort	Journal of the American Academy of Orthopaedic Surgeons (JAAOS)	98 patients, 104 shoulder arthroplasties totalTSA: 52 proceduresrTSA: 52 procedures	TSA: 60.1 ± 7.4 yrsrTSA: 67.5 ± 7.5 yrs	TSA: 29.9 ± 5.1rTSA: 30.0 ± 5.3	TSA: 36/52 = 69.2% malerTSA: 27/52 = 51.9% male	Minimum follow-up reported: 3 moOutcomes/complications reported through 90 d
Chang,^12^ 2025	Impact of systemic lupus erythematosus on adverse outcomes and readmission after total shoulder arthroplasty: a Nationwide Readmission Database analysis 2016â€“2020	United States	Retrospective cohort	Journal of Orthopaedics and Traumatology	Matched sample: 1,960 patients (980 SLE, 980 no-SLE).Weighted to 3,697 hospitalizations (national weights applied).	Mean age (total): 65.71 ± SE 0.24 yrsSLE: 65.70 ± SE 0.32 yrsNo SLE: 65.71 ± SE 0.33 yrsAge-group distribution (n, %; total/SLE/no-SLE):20–60: 426 (23.0)/213 (22.9)/213 (23.1)60–69: 826 (41.4)/413 (41.8)/413 (40.9)70+: 708 (35.6)/354 (35.3)/354 (35.9)		Total: 158/1960 = 8.2% male.SLE: 79/980 = 8.4% male.No SLE: 79/980 = 7.9% male.	30-d and 90-d
Kucharik,^30^ 2022	Comparison of outpatient vs. inpatient anatomic total shoulder arthroplasty: a propensity scoreâ€“matched analysis of 20,035 procedures	United States	Retrospective cohort	JSES International	Total: 20,035 aTSA Inpatient: 18,707Outpatient: 1,328Matched analysis: 5,288 inpatient vs. 1,322 outpatient	Unmatched:<50 yrs: inpatient 2.5%, outpatient 5.2%50–74 yrs: inpatient 67.8%, outpatient 72.3%75–84 yrs: inpatient 25.9%, outpatient 20.6%85+: inpatient 3.8%, outpatient 2.0%Matched:<50 yrs: 4.4% vs. 5.1%50–74 yrs: 74.2% vs. 72.3%75–84 yrs: 19.3% vs. 20.7%85+: 2.2% vs. 2.0%	Unmatched:Normal: inpatient 14.6%, outpatient 18.1%Overweight: 31.9% vs. 36.1%Mildly obese: 27.0% vs. 27.4%Morbidly obese: 25.9% vs. 18.0%Underweight: 0.5% vs. 0.4%Matched:Normal: 17.3% vs. 18.1%Overweight: 37.0% vs. 36.2%Mildly obese: 26.7% vs. 27.5%Morbidly obese: 18.4% vs. 17.9%Underweight: 0.6% vs. 0.4%	Unmatched: inpatient 46.4%, outpatient 57.1%Matched: 57.4% vs. 57.0%	30-d outcomes only
Calkins,^9^ 2022	Safety and short-term outcomes of anatomic vs. reverse total shoulder arthroplasty in an ambulatory surgery center	United States	Retrospective cohort	Journal of Shoulder and Elbow Surgery	Total = 198 (aTSA 117, rTSA 81).	aTSA: 55.7 ± 7.0 yrsrTSA: 61.1 ± 6.0 yrs*P* = .001 (rTSA older).	aTSA: 31.9 ± 5.2.rTSA: 31.3 ± 5.7.	aTSA: 68/117 = 58%.rTSA: 44/81 = 54%.	Final follow-up (mean ± SD): aTSA 2.8 ± 2.2 yrs; rTSA 2.1 ± 1.6 yrs (*P* = .018).90-d follow-up: 100% (all patients).1-yr follow-up: aTSA 95/117 (81%); rTSA 61/81 (75%).
Hachadorian,^25^ 2023	Association between same-day discharge shoulder arthroplasty and risk of adverse events in patients with American Society of Anesthesiologists classification â‰¥3: a cohort study	United States	Retrospective cohort	Journal of Shoulder and Elbow Surgery	Total: 1,814 patients with ASA ≥3• Same-day discharge: 1,005 (55.4%)• Inpatient stay: 809 (44.6%)	• Same-day discharge: 71.4 yrs (SD: 8.3)• Inpatient stay: 72.8 yrs (SD: 8.6)	• Same-day discharge: 31.4 kg/m^2^ (SD: 6.7)• Inpatient stay: 31.8 kg/m^2^ (SD: 6.9)	• Same-day discharge: 50.0% (502/1,005)• Inpatient stay: 42.8% (346/809)	• 90-d postdischarge
Dawes,^16^ 2023	Medicaid payer status is associated with increased 90-day morbidity and resource utilization following primary shoulder arthroplasty: a propensity scoreâ€“matched analysis	United States	Retrospective cohort	Journal of Shoulder and Elbow Surgery	Unmatched: Total 165,810 (control/non-Medicaid 161,147; Medicaid 4,663).Propensity-matched cohorts: control 4,637 vs. Medicaid 4,637 (1:1 match).	Unmatched:Control: 69.81 ± 9.92 yrsMedicaid: 55.29 ± 10.26 yrsMatched:Control: 56.13 ± 11.02 yrsMedicaid: 55.44 ± 10.03 yrs		Unmatched cohortsControl (male): 67,057 (41.6%)Medicaid (male): 2,145 (46.0%)Matched cohortsControl (male): 2,094 (45.2%)Medicaid (male): 2,134 (46.0%)	90 d
Liu,^32^ 2019	Reverse shoulder arthroplasty for proximal humerus fracture: a more complex episode of care than for cuff tear arthropathy	United States	Retrospective cohort	• Journal of Shoulder and Elbow Surgery	• Total: 1,262 patients• CTA: 759• PHFx: 503• Propensity-matched: 503 CTA vs. 503 PHFx = 1,006 total	• CTA unmatched: 70.9 ± 8.7 yrs• PHFx unmatched: 73.6 ± 9.6 yrs (*P* < .001)• CTA matched: 73.0 ± 8.6 yrs• PHFx matched: 73.6 ± 9.6 yrs (*P* = .262)	• CTA unmatched: 30.7 ± 7.2• PHFx unmatched: 30.5 ± 7.5 (*P* < .600)• CTA matched: 30.8 ± 7.4• PHFx matched: 30.5 ± 7.5 (*P* < .568)Obesity I (BMI 30.0–34.9 kg/m^2^)CTA unmatched: 186 (24.4%)PHFx unmatched: 120 (23.9%)CTA matched: 111 (22.1%)PHFx matched: 120 (23.9%)Obesity II (BMI 35.0–39.9 kg/m^2^)CTA unmatched: 109 (14.3%)PHFx unmatched: 62 (12.3%)CTA matched: 74 (14.7%)PHFx matched: 62 (12.3%)Obesity III (BMI ≥40 kg/m^2^)CTA unmatched: 69 (9.1%)PHFx unmatched: 54 (10.7%)CTA matched: 47 (9.3%)PHFx matched: 54 (10.7%)	CTA unmatched: 342/759 males (45.1%)PHFx unmatched: 96/503 males (19.1%)CTA matched: 281/503 males (55.9%)PHFx matched: 96/503 males (19.1%)	• 30 d
Freshman,^21^ 2025	Non-cefazolin antibiotic prophylaxis is associated with increased rates of acute infectious, medical, and surgical complications following primary total shoulder arthroplasty: a database study	United States	Retrospective cohort	JSES Reviews, Reports, and Techniques, 2025	11,646	Mean 69.4 ± 8.8	Mean 30.6 ± 6.3	45.20%	30 d
Joshi,^26^ 2025	Reverse or anatomic shoulder arthroplasty for glenohumeral osteoarthritis in patients over 70: readmission rates and utilization trends	United States	Retrospective cohort	Seminars in Arthroplasty: JSES	251,048 (84,463 aTSAs; 166,585 rTSAs; weighted estimates)	aTSA mean 75.3 yrs; rTSA mean 76.7 yrs		aTSA 44.5% male, rTSA 39.1% male	30 and 90 d (fixed by NRD design; exclusions applied for December admissions)
Haase,^24^ 2025	Comparison study of patient demographics, causes, and risk factors associated with 90-day readmissions following primary reverse shoulder arthroplasty	United States	Retrospective cohort	Seminars in Arthroplasty	116,893 (4,705 re-admitted; 112,188 not re-admitted)			45.50%	90 d
Beauperthuy,^6^ 2025	Reverse Shoulder Arthroplasty in Patients with Rheumatoid Arthritis: A Nationwide Analysis	United States	Retrospective case-control	Journal of Clinical Medicine	43,286 (7,232 RAs; 36,054 controls)	RA 66.8 ± 6.6 yrs		24.40%	90 d (medical), 2 yrs (implant)
Khokhar,^28^ 2024	Does morbid obesity negatively impact perioperative outcomes following elective reverse shoulder arthroplasty?: a propensity-matched comparative study	United States	Retrospective cohort	JSES International	175 (19 morbidly obese vs. 156 non-morbidly obese)	Morbidly obese 68 (IQR: 64–73); non-morbid 71 (IQR: 66.5–77)	Morbidly obese 42.4 (41.0–45.1); non-morbid 30.3 (27.2–33.1)	16%	90 d
Maheshwer,^33^ 2024	Analysis of 90-day complications in outpatient total shoulder arthroplasty	United States	Retrospective cohort	Seminars in Arthroplasty	140 (108 TSAs; 32 rTSAs)	TSA median 57.6 (51.5–62.3); rTSA 62.0 (57.5–66.1)	TSA 30.7 (27.8–35.5); rTSA 31.1 (26.4–36.1)	65% overall (TSA 84%; rTSA 53%)	90 d
Allen,^3^ 2024	Perioperative complications after total shoulder arthroplasty in patients with neurological disorders	United States	Retrospective cohort	Seminars in Arthroplasty	428,761 total (209,789 aTSAs; 218,972 rTSAs); 3,602 (0.8%) had neurologic disorders	TSA 68 ± 9 yrs; rTSA 71 ± 8 yrs		aTSA 45%; rTSA 37%	180 d
Mayfield,^39^ 2024	Reverse total shoulder arthroplasty for proximal humerus fracture: a complex episode of care associated with increased surgical and medical complications	United States	Retrospective cohort	Seminars in Arthroplasty	62,544 total (11,394 PHF; 51,150 OA)	PHF 73.35 ± 8.82 yrs; OA 71.75 ± 8.02 yrs		18%	90 d
Su,^53^ 2024	Impact of surgeon variability on outcomes after total shoulder arthroplasty: an analysis of 2,188 surgeons	United States	Retrospective cohort	Seminars in Arthroplasty	93,132 patients (aTSA 37,957; rTSA 55,175)	aTSA 68.7 ± 8.6 yrs; rTSA 71.2 ± 7.8 yrs		aTSA 47.4%; rTSA 40.2%	2 yrs (complications and revision); 90 d (readmission)
Oeding,^42^ 2023	Understanding risk for early dislocation resulting in reoperation within 90 days of reverse total shoulder arthroplasty: extreme rare event detection through cost-sensitive machine learning	United States	Retrospective cohort	Journal of Shoulder and Elbow Surgery	74,697 (740 dislocations)	69.9 ± 9.3 (dislocation) vs. 72.0 ± 8.5 (no dislocation)		57.3% (dislocation) vs. 37.3% (no dislocation)	90 d
Gordon,^23^ 2023	Interscalene block vs. periarticular liposomal bupivacaine for pain control following reverse shoulder arthroplasty: a randomized trial	United States	Prospective randomized trial	Journal of Shoulder and Elbow Surgery	76 (38 interscalene block; 38 periarticular liposomal bupivacaine)	68 yrs	Block 31.5; local 33.6	55.30%	7–14 d (post-operative appointment)
Devana,^18^ 2021	The influence of prior dental pathology on medical complications and peri-prosthetic joint infections following primary shoulder arthroplasty	United States	Retrospective cohort	European Journal of Orthopaedic Surgery & Traumatology	8,481 (1,419 with dental pathology; 7,062 controls)	Matched by age (most between 55–74 yrs)	Matched by BMI >30 kg/m^2^	45.30%	90 d (medical complications, readmissions); 2 yrs (implant complications)
Deasey,^17^ 2021	Development of a Machine Learning Algorithm for Prediction of Complications and Unplanned Readmission Following Reverse Total Shoulder Arthroplasty	United States	Retrospective cohort	Journal of Shoulder and Elbow Surgery	2,799	69.0 ± 8.9	N/A	51.09%	N/A
Cointat,^14^ 2021	A comparison of perioperative pain control in total shoulder arthroplasty: preoperative tramadol is associated with decreased long-term opiate use when compared with traditional opioids	United States	Retrospective cohort	J Shoulder Arthroplasty	1,099	73.2 ± 8.8	31.0 ± 5.2	19.1% overall male	30 d (short-term)
Wixted,^58^ 2021	Outpatient shoulder prostheses: Feasibility, acceptance and safety	France	Prospective observational cohort	Orthopaedics & Traumatology: Surgery & Research	24	70.0 ± 6.75	26.0 ± 2.75	54%	1 and 6 mo (clinical + radiologic), 90 d (consultation)
Chung,^13^ 2019	Intraoperative fractures in shoulder arthroplasty: risk factors and outcomes	United States	Retrospective cohort	JSES International	1,773 (994 anatomic, 779 reverse)	Fracture cohort: 72.09 yrs; non-fracture: 68.62 yrs	Fracture cohort: 31.90; non-fracture: 30.35	Fracture cohort: 19% male (81% female)	N/A
Anakwenze,^4^ 2017	Analysis of 90-Day Readmissions After Total Shoulder Arthroplasty	United States	Retrospective cohort	J Shoulder Elbow Surg	26,023	67.3 ± 9.5 (non-re-admitted), 66.2 ± 9.3 (re-admitted)	N/A	48.60%	90 d
Streubel,^52^ 2014	Effect of Age on Outcomes of Shoulder Arthroplasty	United States	Retrospective cohort	The Permanente Journal	2,575 arthroplasties: 2,007 total shoulder arthroplasties (TSAs) and 568 reverse TSAs (rTSAs)	Older cohort 79 yrs (77–82) vs. younger 66 yrs (61–71)	NR	38.3% of the older group vs. 54.7% of the younger	90-d
Cullen,^15^ 2024	Thirty and ninety-day reoperation rates after shoulder arthroplasty	United States	Retrospective cohort	The Journal of Bone and Joint Surgery	2,305 primary shoulder arthroplasties and 518 revision shoulder arthroplasties	68	30.3	46	30 and 90 d
Kiet,^29^ 2015	Is the use of abduction pillows crucial for post-operative rehabilitation following reverse total shoulder arthroplasty?	United States	Retrospective cohort	Shoulder & Elbow	183 reverse total shoulder arthroplasties (rTSAs): 107 with abduction pillow vs. 76 without	70 ± 8 yrs		40	90 d
Merolla,^40^ 2020	Outcomes after shoulder replacement: comparison between reverse and anatomic total shoulder arthroplasty	United States, single tertiary-care institution	Prospective case-control	Journal of Shoulder and Elbow Surgery	TSA: 47, rTSA: 53				TSA & rTSA: 2.1 ± 0.8 yrs
Parada,^44^ 2021	Pre-operative factors affecting the indications for anatomical and reverse total shoulder arthroplasty in primary osteoarthritis and outcome comparison in patients aged seventy years and older	Italy, multicenter (4 shoulder units)	Prospective cohort	International Orthopaedics, Volume 44 (2020)	TSA: 47; rTSA: 36 (total shoulders: 83; sub-group ≥70 yrs: TSA 26, rTSA 32)	Mean age overall: 71.6 yrs (range, 68–72); TSA: mean 70 yrs (68–72); rTSA: mean 74 yrs (69–75)	BMI: not reported numerically; no significant difference between TSA and rTSA	Male: 19% (overall); female: 77%	Mean follow-up: 28.8 mo (27–30); TSA: 29 mo (SD: 1.3); rTSA: 28.5 mo (SD: 4.5)
Aibinder,^2^ 2021	Comparison of complication types and rates associated with anatomic and reverse total shoulder arthroplasty	United States & Europe, multicenter database	Retrospective cohort	Journal of Shoulder and Elbow Surgery, 2020	aTSA: 2,224, rTSA: 4,158	aTSA: 66 ± 9, rTSA: 72 ± 8	aTSA: 30 ± 6, rTSA: 29 ± 6	aTSA: 49%, rTSA: 36%	aTSA: 34 mo, rTSA: 22 mo
Casp,^10^ 2020	Risk factors for complications and revision surgery after anatomic and reverse total shoulder arthroplasty	International, multicenter database (United States & Europe)	Retrospective cohort	Journal of Shoulder and Elbow Surgery, 2021	Total 8,580 (aTSA: 2,964, rTSA: 5,616)	aTSA: 66 ± 9.1, rTSA: 72 ± 7.8	aTSA: 30.1 ± 6.3, rTSA: 28.9 ± 6.0	aTSA: 49.7%, rTSA: 37.1%	aTSA: 38 ± 35.8 mo, rTSA: 24 ± 24.7 mo
Flurin,^20^ 2020	Osteoporosis and Implant-Related Complications After Anatomic and Reverse Total Shoulder Arthroplasty	United States, National Medicare Database, 2005-2014	Retrospective cohort	Journal of the American Academy of Orthopaedic Surgeons, 2020, 28(3):121-127	TSA osteoporosis: 17,078; rTSA osteoporosis: 17,287	Not specified; age distribution provided in Tables I and II	BMI not reported individually; obesity TSA: 18.0%, morbid obesity 12.2%; rTSA: 17.7%, morbid obesity 11.4%	TSA % male: 10.7% (ie, 89.3% female); rTSA % Male: 9.4% (90.6% female)	Minimum 2-yr follow-up
Friedman,^22^ 2022	Comparison of survivorship and performance of a platform shoulder system in anatomic and reverse total shoulder arthroplasty	Multinational (United States, United Kingdom, Europe), multisurgeon registry	Prospective cohort	Journal not explicitly stated; PMID: 33345236	aTSA: 286, rTSA: 492	aTSA: 67.8 ± 9.6 yrs, rTSA: 74.2 ± 7.4 yrs	aTSA: 29.4 ± 15.0, rTSA: 28.1 ± 12.5	TSA: 44% male, rTSA: 32% male	aTSA: 64.0 ± 32.0 mo, rTSA: 56.4 ± 23.8 mo
Liu,^31^ 2022	Comparison of Reverse and Anatomic Total Shoulder Arthroplasty in Patients With an Intact Rotator Cuff and No Previous Surgery	International multi-institutional (United States & Europe) registry	Retrospective cohort	Journal of the American Academy of Orthopaedic Surgeons, 2022, 30(19):941-948	aTSA: 370, rTSA: 370	Mean 73 yrs (both groups)	BMI 29 (both groups)	aTSA: 39%; rTSA: 39% male	Mean follow-up aTSA: 41 mo, rTSA: 42 mo
Nazzal,^41^ 2023	Total shoulder arthroplasty vs. reverse total shoulder arthroplasty: Outcome comparison in osteoarthritis patients with or without concurrent rotator cuff deficiency	United States, Nationwide Inpatient Sample (NIS) database	Retrospective cohort	Journal: Not specified (PMCID: PMC9371544)	TSA: 25,554, rTSA: 22,337	Age TSA: 67.31 ± 9.66 yrs, rTSA: 72.38 ± 8.93 yrs	BMI: not reported	TSA male: 49.21%, rTSA male: 36.21%	Follow-up: inpatient episode only
Trammell,^54^ 2023	Reverse shoulder arthroplasty with preservation of the rotator cuff for primary glenohumeral osteoarthritis has similar outcomes to anatomic total shoulder arthroplasty and reverse shoulder arthroplasty for cuff arthropathy	United States, single tertiary-care institution (UPMC Freddie Fu Sports Medicine Center, Pittsburgh, PA; University of Nebraska Medical Center, Omaha, NE)	Retrospective cohort	Journal of Shoulder and Elbow Surgery, 2023	+rcRSA: 24; –rcRSA: 69; TSA: 93	þrcRSA: 71.1 ± 6.9 yrs; –rcRSA: 72.4 ± 8.7 yrs; TSA: 66.0 ± 6.2 yrs	þrcRSA: 31.8 ± 5.2; –rcRSA: 31.1 ± 4.6; TSA: 31.0 ± 4.5	þrcRSA: 24.2% male; –rcRSA: 62.3% male; TSA: 62.4% male	Mean follow-up: þrcRSA 16.2 mo; –rcRSA 19.7 mo; TSA 17.7 mo
Villacis,^56^ 2016	Clinical outcomes of anatomical vs. reverse total shoulder arthroplasty in patients with primary osteoarthritis, an intact rotator cuff, and limited forward elevation	United States; single tertiary academic center (University of Florida, Gainesville)	Prospective cohort	Bone & Joint Journal 2023;105-B(12):1303–1313	507 total (344 aTSAs; 163 rTSAs) with ≥2-yr follow-up	aTSA stiff: 65.0 ± 7.2 yrs; aTSA non-stiff: 65.7 ± 7.1 yrs; rTSA stiff: 71.7 ± 7.0 yrs; rTSA non-stiff: 71.4 ± 6.3 yrs	aTSA stiff: 32.1 ± 7.2 kg/m^2^; aTSA non-stiff: 30.8 ± 7.1; rTSA stiff: 30.9 ± 6.3; rTSA non-stiff: 31.2 ± 6.7	aTSA: 41.2% male; rTSA: 50.0% male	Mean follow-up: aTSA 5.9 ± 3.3 yrs; rTSA 3.5 ± 1.7 yrs
Ponce,^46^ 2015	Complication rate and implant survival for reverse shoulder arthroplasty vs. total shoulder arthroplasty: results during the initial 2 years	United States (California), Statewide OSHPD database, inpatient + ambulatory + ED settings	Retrospective cohort	Journal of Shoulder and Elbow Surgery, 2016	Total: 10,844 (TSA: 6,658; rTSA: 4,186)	TSA: 68.1 ± 9.8 yrs; rTSA: 73.3 ± 9.0 yrs	BMI not reported	TSA: 49% male; rTSA: 64% male	Up to 2 yrs post-operative (30 d, 90 d, 1 yr, 2 yrs)
Mahoney,^34^ 2014	Comparative analysis of anatomic and reverse total shoulder arthroplasty: in-hospital outcomes and costs	United States, Nationwide Inpatient Sample (NIS), multicenter national database	Retrospective cohort	Journal of Shoulder and Elbow Surgery (2015)	Total: 51,052 (aTSA: 29,359; rTSA: 21,693)	Overall mean age 69 ± 12 yrs; aTSA 67 ± 12 vs. rTSA 73 ± 11	Not reported	aTSA: 50%; rTSA: 36%	In-hospital only (index admission)
Burnett,^8^ 2022	Readmission after shoulder arthroplasty	United States, New York, single tertiary academic center (NYU Langone Medical Center)	Retrospective cohort	Journal of Shoulder and Elbow Surgery (2014)	680 (HA: 124; TSA: 376; rTSA: 180)	Not reported	Not reported	Not reported	90-d post-operative period
Valsamis,^55^ 2024	Characteristics and risk factors for 90-day readmission following shoulder arthroplasty	United States, bi-institutional tertiary referral centers (Rush University Medical Center, Chicago IL; Duke University Medical Center, Durham NC)	Retrospective cohort	Journal of Shoulder and Elbow Surgery (2022), Vol 31, pp. 324–332	Total: 5,410 (aTSA: 2,805; rTSA: 2,605)	Mean: 66.9 yrs (surgical readmission), 70 yrs (medical readmission)	Mean: 31.1 (surgical), 30.6 (medical)	48%	90-d post-operative follow-up
Valsamis^55^	Reverse total shoulder replacement vs. anatomical total shoulder replacement for osteoarthritis: population based cohort study using data from the National Joint Registry and Hospital Episode Statistics for England	United Kingdom	Retrospective registry-based cohort	BMJ	Matched cohort: 7,124 (TSA: 3,562, rTSA: 3,562); weighted cohort: 12,968 (TSA: 9,393, rTSA: 3,575)	Matched: TSA 76.9 ± 6.2, rTSA 77.0 ± 6.3; weighted: TSA 73.8 ± 7.0, rTSA 73.9 ± 6.7		TSA 23.0%, rTSA 22.5% (matched cohort)	Maximum 8.75 yrs

*SD*, standard deviation; *IQR*, interquartile range; *BMI*, body mass index; *ASC*, ambulatory surgical center; *HSC*, hospital-based surgical center; *aTSA*, anatomic total shoulder arthroplasty; *rTSA*, reverse total shoulder arthroplasty; *TSA*, total shoulder arthroplasty; *SLE*, systemic lupus erythematosus; *ASA*, American Society of Anesthesiologists; *PHFx*, proximal humerus fracture; *NRD*, National Readmissions Database; *OA*, osteoarthritis; *N/A*, not applicable; *NR*, not reported; *ED*, emergency department; *OSHPD*, California Office of Statewide Health Planning and Development; *HA*, hemiarthroplasty; *PMID*, PubMed Identifier; *PMCID*, PubMed Central Identifier; *SE,* standard error; *JSES*, *Journal of Shoulder and Elbow Surgery*; *CTA,* computed tomography angiography; *RA,* rheumatoid arthritis.

**Table II:** Summary of included studies.

Author (yr)	Title	Data source	Aims/methods	Conclusion
Mastrokostas,^36^ 2025	Outcomes of ambulatory vs. outpatient hospital-based surgical center shoulder arthroplasty: complications, readmissions, and charges	PearlDiver Mariner	Compared outpatient TSA in ASCs vs. HSCs for complications, re-admissions, and costs. Used matched cohorts and regression analyses to assess outcomes and cost differences.	ASCs provide similar safety with lower complication rates and markedly lower costs, although with slightly higher re-admissions. Overall, ASCs are a cost-effective alternative for outpatient TSA.
Mayfield,^37^ 2025	Effect of body mass index on complications following total shoulder arthroplasty: a population-wide analysis	Premier	To evaluate whether higher BMI influences surgical and medical complications after anatomic and reverse shoulder arthroplasty, comparing outcomes across BMI groups using post-operative complication data.	Higher BMI did not increase surgical complication rates, but BMI >35 kg/m^2^ was linked to more medical issues post-rTSA particularly respiratory and renal complications highlighting the need for careful counseling and selection of obese patients.
Marigi,^35^ 2023	Higher rates of mortality and perioperative complications in patients undergoing primary shoulder arthroplasty and a history of previous stroke	Institutional registry	To determine how a prior stroke with ongoing sequela affects outcomes after primary shoulder arthroplasty. It compared post-operative complications, re-admissions, and mortality between patients with previous stroke and matched controls using long-term follow-up.	Patients with a history of stroke experienced substantially higher surgical and medical complications, including instability, VTE, respiratory issues, re-admissions, and long-term mortality. These elevated risks highlight the need for careful counseling and perioperative optimization in this group.
Seetharam,^49^ 2022	Trends in outpatient shoulder arthroplasty during the COVID-19 (coronavirus disease 2019) era: increased proportion of outpatient cases with decrease in 90-day readmissions	Single institution	To assess how the COVID-19 era affected outpatient shoulder arthroplasty and post-operative outcomes. It reviewed all primary anatomic and reverse TSAs over 3 yr, comparing pre-COVID and during-COVID periods while matching patient characteristics to evaluate outpatient rates, length of stay, and 90-d complications.	Outpatient TSA increased substantially during COVID-19, with shorter hospital stays and fewer 90-d readmissions, while other complication rates remained unchanged. The findings indicate that outpatient TSA can be delivered safely and efficiently in appropriately selected patients.
Antonacci,^5^ 2021	Complications and Readmissions after Reverse and Anatomic Total Shoulder Arthroplasty with Same-day Discharge	Single institution	To compare 90-d complication and readmission rates between anatomic and reverse total shoulder arthroplasty with same-day discharge. Patients were selected for outpatient surgery using standardized criteria, and outcomes including demographics, operative time, complications, and readmissions were compared between the 2 groups.	Reverse total shoulder arthroplasty with same-day discharge is safe for appropriately selected patients, with 90-d complication and readmission rates similar to those of anatomic total shoulder arthroplasty.
Chang,^12^ 2025	Impact of systemic lupus erythematosus on adverse outcomes and readmission after total shoulder arthroplasty: a Nationwide Readmission Database analysis 2016â€“2020	NRD	To assess the impact of systemic lupus erythematosus (SLE) on short-term outcomes after total shoulder arthroplasty (TSA) by comparing patients with and without SLE and using propensity-score matching based on age and sex.	SLE increases the risk of post-operative complications and 30-d and 90-d re-admissions after TSA, particularly in older patients and those undergoing reverse TSA, although lack of data on immunosuppressive therapy and disease activity limits the strength of the evidence.
Kucharik,^30^ 2022	Comparison of outpatient vs. inpatient anatomic total shoulder arthroplasty: a propensity scoreâ€“matched analysis of 20,035 procedures	ACS-NSQIP	To evaluate the clinical outcomes of outpatient vs. inpatient anatomic total shoulder arthroplasty (aTSA) by comparing 30-d adverse events, re-admissions, and operative times using a propensity score-matched analysis.	Outpatient aTSA was associated with fewer serious adverse events and similar re-admission rates compared with inpatient aTSA, supporting its safe use in appropriately selected patients.
Calkins,^9^ 2022	Safety and short-term outcomes of anatomic vs. reverse total shoulder arthroplasty in an ambulatory surgery center	2-ASC review	To compared early post-operative outcomes between outpatient anatomic and reverse total shoulder arthroplasty and assessed whether adding a pre-operative interscalene nerve block to periarticular liposomal bupivacaine improved recovery in the postanesthesia care unit.	Early outcomes and complication rates were similar between the procedures, all patients were discharged the same day, and pre-operative nerve blocks reduced PACU pain, opioid use, and discharge time.
Hachadorian,^25^ 2023	Association between same-day discharge shoulder arthroplasty and risk of adverse events in patients with American Society of Anesthesiologists classification â‰¥3: a cohort study	Kaiser Permanente	To compare outcomes of same-day discharge vs. inpatient shoulder arthroplasty in patients with higher comorbidity (ASA ≥3), focusing on 90-d post-discharge events. Propensity score-weighted logistic regression was used to assess non-inferiority for re-admission, complications, and other adverse events.	Same-day discharge did not increase the likelihood of readmissions or complications compared with an inpatient stay and was non-inferior for these outcomes, suggesting it may be safely expanded to higher-risk patients.
Dawes,^16^ 2023	Medicaid payer status is associated with increased 90-day morbidity and resource utilization following primary shoulder arthroplasty: a propensity scoreâ€“matched analysis	NRD	To examine whether Medicaid payer status affects 90-d outcomes and resource utilization after primary shoulder arthroplasty (aTSA, rTSA, HA). Using the National Readmission Database (2011–2016), Medicaid vs. non-Medicaid patients were identified, then matched 1:1 using propensity scores to compare 90-d re-admission, re-operation, length of stay, and direct costs.	Medicaid payer status was independently associated with higher 90-d all-cause and shoulder-related readmissions, re-operations, longer length of stay, and increased direct costs after primary shoulder arthroplasty, indicating greater resource utilization and the need for risk adjustment to ensure fair access and reimbursement.
Liu,^32^ 2019	Reverse shoulder arthroplasty for proximal humerus fracture: a more complex episode of care than for cuff tear arthropathy	NSQIP	To compared in-hospital and 30-d post-operative outcomes of reverse total shoulder arthroplasty performed for proximal humerus fracture vs. cuff tear arthropathy using NSQIP data. After 1:1 propensity matching on demographics and comorbidities, operative time, length of stay, complications, transfusion, re-admission, and discharge destination were analyzed.	rTSA for proximal humerus fracture was associated with significantly higher complication rates, greater transfusion need, longer hospital stay, and increased discharge to extended care facilities compared with rTSA for cuff tear arthropathy, indicating higher resource utilization in fracture patients.
Freshman,^21^ 2025	Non-cefazolin antibiotic prophylaxis is associated with increased rates of acute infectious, medical, and surgical complications following primary total shoulder arthroplasty: a database study	Premier	This retrospective cohort study used the Premier Healthcare Database (2016-2020) to identify adults undergoing primary elective shoulder arthroplasty and classify them by perioperative prophylactic antibiotic regimen (cefazolin, clindamycin, vancomycin, or combinations). Cefazolin monotherapy served as the reference group, and multivariate logistic regression evaluated 90-d risks of post-operative complications including periprosthetic joint infection and acute kidney injury.	Clindamycin and vancomycin monotherapy were associated with higher rates of periprosthetic joint infection, and vancomycin (alone or combined) increased acute kidney injury risk; cefazolin monotherapy demonstrated the lowest complication risk and is supported as the preferred prophylactic regimen for primary total shoulder arthroplasty.
Joshi,^26^ 2025	Reverse or anatomic shoulder arthroplasty for glenohumeral osteoarthritis in patients over 70: readmission rates and utilization trends	NRD	This study used the Nationwide Readmissions Database (2016-2021) to identify patients aged ≥70 undergoing anatomic or reverse TSA for glenohumeral osteoarthritis, comparing 30-d and 90-d readmission rates and causes of re-admission, while also analyzing national trends in arthroplasty utilization.	Patients aged ≥70 who underwent rTSA had higher 30-d and 90-d readmission risks compared with aTSA, and rTSA use for GHOA has continued to rise steadily at approximately 3.8% per year.
Haase,^24^ 2025	Comparison study of patient demographics, causes, and risk factors associated with 90-day readmissions following primary reverse shoulder arthroplasty	Nationwide Admin DB (Likely NRD)	This national administrative database study identified patients who underwent reverse total shoulder arthroplasty (rTSA). Ninety-day readmission rates were calculated, and the readmitted cohort was compared with non-readmitted controls. Patient demographics, comorbidities, and Elixhauser Comorbidity Index (ECI) were analyzed using chi-square testing. Multivariate logistic regression was performed to determine patient-related risk factors associated with 90-d readmission.	Ninety-day readmission after rTSA occurred in 4% of patients, with prosthetic dislocation and surgical site infection being the most common causes. Severe obesity, rheumatoid arthritis, iron-deficiency anemia, chronic kidney disease, and higher ECI scores significantly increased re-admission risk. These findings highlight the importance of recognizing high-risk comorbidities to optimize pre-operative counseling and risk-stratification in rTSA candidates.
Beauperthuy,^6^ 2025	Reverse Shoulder Arthroplasty in Patients with Rheumatoid Arthritis: A Nationwide Analysis	PearlDiver - 170Ortho	This retrospective case-control study used a national administrative claims database (2010-2023) to evaluate the effect of rheumatoid arthritis (RA) on post-operative outcomes following reverse shoulder arthroplasty (rTSA). Patients with RA undergoing rTSA were identified using diagnostic and procedural codes and were propensity score matched 1:5 to non-RA rTSA controls. Medical complications within 90 d, implant-related complications within 2 y, fall rates, and healthcare utilization—including length of stay—were compared between groups.	RA was associated with substantially higher post-operative risk after rTSA. Compared with matched controls, RA patients demonstrated markedly elevated 90-d medical complications particularly UTI, pulmonary embolism, and blood transfusion need as well as significantly increased 2-y implant-related complications. RA patients also experienced higher fall rates and longer hospital stays. These findings indicate RA is an independent risk factor for adverse outcomes following rTSA, highlighting the need for enhanced perioperative optimization and closer post-operative monitoring to reduce complications, LOS, and healthcare burden.
Khokhar,^28^ 2024	Does morbid obesity negatively impact perioperative outcomes following elective reverse shoulder arthroplasty?: a propensity-matched comparative study	Single tertiary center	This single-center retrospective study reviewed consecutive elective primary rTSA cases (2016–2023) using the same short-stem humeral implant and screw-in glenoid system. Patients with morbid obesity (BMI ≥40) were propensity matched ≥1:1 to non-morbidly obese patients based on age, sex, mFI-5, adjusted Charlson Comorbidity Index, and 12-mo pre-operative ED utilization. Regression analysis assessed the effect of morbid obesity on operative time, blood loss, length of stay, transfusion need, post-operative surgical and medical complications, discharge disposition, and 90-d ED return or readmission.	Morbidly obese patients (BMI ≥40) had no significant differences in operative measures, post-operative complications, hospital stay, ED returns, or readmission compared with matched non-morbidly obese patients. These findings indicate that morbid obesity alone should not be considered a contraindication to elective rTSA when patients receive appropriate pre-operative optimization.
Maheshwer,^33^ 2024	Analysis of 90-day complications in outpatient total shoulder arthroplasty	Institutional registry	The study aimed to evaluate complications, ER visits, and readmissions following outpatient total shoulder arthroplasty (TSA) and reverse TSA (rTSA). A retrospective analysis of a prospectively maintained registry was performed on patients who underwent outpatient TSA or rTSA between 2016 and 2019, with 90-d post-operative outcomes assessed.	Outpatient TSA and rTSA demonstrated low rates of complications, ER visits, and no readmissions or reoperations. Higher body mass index was associated with increased complication risk, supporting the safety of outpatient procedures in selected patients.
Allen,^3^ 2024	Perioperative complications after total shoulder arthroplasty in patients with neurological disorders	NRD	The study aimed to evaluate perioperative and early post-operative outcomes in patients with neurologic disorders undergoing total shoulder arthroplasty (TSA). Using the Nationwide Readmission Database, 428,761 TSA cases from 2011–2019 were analyzed, with 3,602 patients identified as having neurologic conditions; statistical analyses included ANOVA, Chi-squared/Fisher's exact tests, and logistic regression adjusted for demographics and comorbidities.	Patients with neurologic disorders undergoing TSA face higher risks of perioperative complications, extended hospital stays, non-home discharge, and 180-d mortality. Recognizing these patients pre-operatively can help tailor management strategies to improve both short-term and long-term outcomes.
Mayfield,^39^ 2024	Reverse total shoulder arthroplasty for proximal humerus fracture: a complex episode of care associated with increased surgical and medical complications	Premier	The study aimed to compare demographics, comorbidities, and 90-d outcomes between patients undergoing reverse total shoulder arthroplasty (rTSA) for proximal humerus fractures (PHF) vs. osteoarthritis (OA). Using the Premier Healthcare Database (2016-2020), PHF patients were matched 2:1 with OA controls based on age, sex, race, and comorbidities, and surgical/medical complications and re-admissions were analyzed using descriptive statistics and regression.	Patients receiving rTSA for PHF have significantly higher risks of surgical and medical complications and 90-d readmission compared to OA controls. These results highlight the need for careful pre-operative risk assessment to reduce patient morbidity.
Su,^53^ 2024	Impact of surgeon variability on outcomes after total shoulder arthroplasty: an analysis of 2188 surgeons	PearlDiver Mariner	The study aimed to assess how surgeon-specific factors such as procedural volume, career duration, practice type, and fellowship training affect outcomes after total shoulder arthroplasty (TSA). Using the PearlDiver Mariner database, 2,188 surgeons performing ≥11 TSAs were analyzed, and multivariate logistic regression evaluated relationships between surgeon variables and 2-yr complications, revisions, and 90-d re-admissions.	Surgeon factors like procedural volume, career length, and practice setting do influence complications, revisions, and readmissions after TSA, but their impact is smaller than patient-specific factors. This emphasizes that careful patient selection is critical to reducing adverse outcomes.
Oeding,^42^ 2023	Understanding risk for early dislocation resulting in reoperation within 90 days of reverse total shoulder arthroplasty: extreme rare event detection through cost-sensitive machine learning	NRD	The study aimed to predict 90-d readmission due to dislocation after reverse total shoulder arthroplasty using machine learning. A retrospective review of 74,697 patients from the Nationwide Readmissions Database was conducted, evaluating 5 algorithms and using SHAP values and hierarchical clustering to assess risk factors and patient cohorts.	Machine learning reliably identified patients at risk for 90-d dislocation-related readmission after rTSA, with young, male patients with high comorbidity burdens post-humerus fracture being particularly vulnerable. These findings highlight the need for tailored post-operative management and counseling to reduce dislocation risk.
Gordon,^23^ 2023	Interscalene block vs. periarticular liposomal bupivacaine for pain control following reverse shoulder arthroplasty: a randomized trial	RCT (primary data)	This randomized study of adults undergoing reverse shoulder arthroplasty compared pre-operative interscalene nerve blocks with intraoperative periarticular liposomal bupivacaine injections. Primary outcomes included narcotic consumption, pain scores, and patient satisfaction, while secondary outcomes assessed psychosocial factors, patient preferences, and post-operative resource use.	Interscalene nerve blocks resulted in higher patient satisfaction than liposomal bupivacaine injections, with no significant differences in pain scores, narcotic use after day 0, re-admissions, or other post-operative metrics. Psychosocial factors and patient preferences did not influence satisfaction.
Devana,^18^ 2021	The influence of prior dental pathology on medical complications and peri-prosthetic joint infections following primary shoulder arthroplasty	PearlDiver (datasent unspecified)	The study aimed to assess the impact of prior dental pathology on outcomes after shoulder arthroplasty, including LOS, medical complications, readmissions, implant-related complications, and healthcare costs. Using the PearlDiver database, 1,419 patients with prior dental pathology were matched 1:5 to 7,062 controls by age, sex, and comorbidities, and outcomes were analyzed with logistic regression and *t*-tests.	Patients with prior dental pathology experienced higher 90-d medical complications and 2-yr implant-related issues, but similar rates of PJIs and healthcare costs. These results can guide surgeons in counseling patients with relevant dental histories.
Deasey,^17^ 2021	Development of a Machine Learning Algorithm for Prediction of Complications and Unplanned Readmission Following Reverse Total Shoulder Arthroplasty	California Office of Statewide Health Planning and Development (OSHPD) database	To develop and validate a machine-learning model to predict major post-operative complications or 30-d readmission following primary reverse total shoulder arthroplasty (rTSA). Using a retrospective chort of patients from California (2015–2017), the authors trained and compared multiple classifiers including logistic regression, XGBoost, and random forest across 64 patient-specific and procedure-specific variables, assessing performance via AUROC, AUPRC, and Brier scores	XGBoost achieved the highest predictive accuracy (AUROC 0.681, AUPRC 0.129), identifying prior implant complications, protein-calorie malnutrition, and higher comorbidity burden as key predictors, demonstrating that machine learning can effectively stratify early post-operative risk in rTSA.
Cointat,^14^ 2021	A comparison of perioperative pain control in total shoulder arthroplasty: pre-operative tramadol is associated with decreased long-term opiate use when compared with traditional opioids	PearlDiver Patient Records Database	This retrospective cohort study used a private insurance database to compare perioperative pain management in total and reverse shoulder arthroplasty patients between 2008 and 2015. Patients were grouped by analgesic regimen traditional opioids, tramadol, or non-opioids and outcomes including length of stay, 90-d readmissions, ED visits, medical complications, and prolonged opioid use (>3 mo) were evaluated.	Perioperative tramadol was associated with a lower risk of prolonged opioid use compared with traditional opioids, without increasing medical complications, ED visits, or readmissions. These findings support consideration of tramadol for shoulder arthroplasty candidates, while acknowledging that opioid-free regimens remain safest for minimizing prolonged post-operative narcotic use.
Wixted,^58^ 2021	Outpatient shoulder prostheses: Feasibility, acceptance and safety	Single-center clinical/hospital data (Clinicom V2019 and Ortho+ V17), supplemented by prospectively collected telephone interviews, patient-reported data and clinical follow-up	To assess early morbidity and mortality after outpatient shoulder arthroplasty to validate safety and eligibility criteria. To assess patient acceptance of outpatient shoulder arthroplasty.	Outpatient shoulder arthroplasty was safe in carefully selected low-comorbidity patients, with no reported complications or readmissions. However, patient acceptance was low, and many declined the outpatient pathway, indicating a need for better patient education.
Chung,^13^ 2019	Intraoperative fractures in shoulder arthroplasty: risk factors and outcomes	Single-center institutional shoulder arthroplasty database and electronic medical records from a single tertiary referral health system	To determine the incidence of intraoperative fractures during shoulder arthroplasty and identify patient-related or procedure-related risk factors, as well as assess short-term outcomes such as length of stay and 90-d readmission.	Intraoperative fractures were rare (1.2%) but occurred more frequently in reverse arthroplasty, revision cases, female patients, and those with liver disease. Fractures were associated with a longer hospital stay, though increased 90-d readmission and SNF/rehab discharge rates were not statistically significant. These findings support improved pre-operative counseling and shared decision-making.
Anakwenze,^4^ 2017	Analysis of 90-d Readmissions After Total Shoulder Arthroplasty	National Readmissions Database (NRD)	To characterize national 90-d readmission rates after primary anatomic TSA, identify risk factors and causes for readmissions, and assess associated healthcare resource utilization.	Ninety-day readmissions were uncommon (1.7%) but costly and more frequent than 30-d events. Predictors included Medicare insurance, COPD, and discharge to skilled nursing facilities, while female sex was protective. Hardware-related complications were the most common cause of readmission. Limiting readmission analysis to 30 d misses most TSA-related readmissions.
Streubel,^52^ 2014	Effect of Age on Outcomes of Shoulder Arthroplasty	Kaiser Permanente Shoulder Arthroplasty Registry	To evaluate how age (>75 vs. ≤75) affects revision rates, mortality, and hospital readmissions after total shoulder arthroplasty (TSA) and reverse total shoulder arthroplasty (rTSA).	Age had different effects in TSA vs. rTSA. In TSA, older patients had higher 1-yr mortality and higher 90-d readmission rates. In rTSA, older patients had lower revision risk. Age should be considered in pre-operative counseling, recognizing distinct risks for TSA vs. rTSA.
Cullen,^15^ 2024	Thirty and 90-day reoperation rates after shoulder arthroplasty	Mayo Clinic Joint Registry	To determine 30-d and 90-d reoperation rates after primary and revision shoulder arthroplasty from 2000–2010, and to identify common causes of reoperation.	Short-term reoperation was uncommon (0.6% at 30 d, 1.4% at 90 d for primary TSA; higher for revisions). Instability and infection were the most frequent causes. Reoperation was approximately twice as common after revision procedures, and most reoperations required hospital readmission.
Kiet,^29^ 2015	Is the use of abduction pillows crucial for post-operative rehabilitation following reverse total shoulder arthroplasty?.	Single-center institutional clinical records from a single fellowship-trained shoulder surgeon	To compare post-operative outcomes of reverse total shoulder arthroplasty (rTSA) in patients using a sling with vs. without an abduction pillow, focusing on dislocation rates, range of motion, and patient-reported outcomes.	Using a sling without an abduction pillow was safe and did not increase risks of dislocation, infection, readmission, revision, or reoperation. Range of motion was actually better in the no-pillow group early post-operative, and patient-reported outcomes were similar.
Merolla,^40^ 2020	Outcomes after shoulder replacement: comparison between reverse and anatomic total shoulder arthroplasty	Single institution	This prospective case-control study compared outcomes and complications between patients undergoing anatomic TSA for glenohumeral arthritis and patients undergoing RTSA for rotator cuff tear arthropathy, with >2 y of follow-up. Major complications, revision rates, patient-reported outcomes (ASES score, VAS), range of motion, and radiographic changes were assessed.	TSA and rTSA demonstrated similar complication and revision rates, patient-reported outcomes, and most ranges of motion at 2 yrs, although TSA provided superior external rotation. These findings support comparable short-term safety and functional outcomes between the 2 procedures.
Parada,^44^ 2021	Pre-operative factors affecting the indications for anatomical and reverse total shoulder arthroplasty in primary osteoarthritis and outcome comparison in patients aged seventy years and older	Multicenter institutional database	To evaluated pre-operative factors influencing the choice of anatomical (TSA) vs. reverse total shoulder arthroplasty (rTSA) in patients ≥70 yrs with primary osteoarthritis and compared post-operative outcomes. Fifty-eight patients underwent TSA or rTSA, with assessments of shoulder range of motion, pain, Constant Murley score, rotator cuff fatty infiltration, glenoid morphology, and radiographic outcomes pre- and post-operatively.	rTSA patients were older, had poorer pre-operative mobility, and greater rotator cuff fatty infiltration than TSA patients. Both procedures achieved similar mid-term improvements in function and range of motion, though TSA showed higher lateral elevation and radiographic changes.
Aibinder,^2^ 2021	Comparison of complication types and rates associated with anatomic and reverse total shoulder arthroplasty	International shoulder arthroplasty database of the Equinoxe (Exactech) single-platform system – 40 surgeons across the USA and Europe	This study used an international database of primary shoulder arthroplasties performed by 40 surgeons to compare the types, incidence, and timing of complications and revisions associated with a single-platform aTSA and rTSA system. Adverse events and revisions were analyzed to identify the most common failure modes for each procedure.	Overall complication rates were 10.7% for aTSA and 8.9% for rTSA, with revision rates of 5.6% and 2.5%, respectively. The predominant complications were procedure-specific (aTSA: rotator cuff/subscapularis failure, aseptic glenoid loosening; rTSA: acromial/scapular fractures, instability), while infection rates were similar, highlighting targets for prosthesis and technique improvement.
Casp,^10^ 2020	Risk factors for complications and revision surgery after anatomic and reverse total shoulder arthroplasty	International prospective shoulder arthroplasty database	To evaluated patient-, implant-, and technique-specific risk factors for intraoperative and post-operative complications, as well as revision surgery in patients from an international primary shoulder arthroplasty database, using multivariate analyses to quantify associated risks.	Younger age, previous shoulder surgery, rheumatoid arthritis, and specific implant characteristics increased the risk of complications and revision. These findings help guide patient counseling and set realistic expectations following aTSA or rTSA.
Flurin,^20^ 2020	Osteoporosis and Implant-Related Complications After Anatomic and Reverse Total Shoulder Arthroplasty	PearlDiver Patient Records	To determine the prevalence of osteoporosis in patients undergoing anatomic and reverse total shoulder arthroplasty (TSA) and assess its impact on prosthetic complications. Complication rates, including periprosthetic fracture, dislocation, loosening/osteolysis, and revision, were compared between patients with and without osteoporosis using multivariable logistic regression to control for demographics, comorbidities, and surgical indication.	Osteoporosis was common in both aTSA and rTSA patients and independently increased the risk of periprosthetic fracture and revision within 2 yrs, highlighting the need for pre-operative counseling and tailored risk mitigation strategies in this population.
Friedman,^22^ 2022	Comparison of survivorship and performance of a platform shoulder system in anatomic and reverse total shoulder arthroplasty	Multinational shoulder arthroplasty registry with prospectively collected data	This study prospectively compared midterm survivorship, clinical outcomes, and complication profiles of primary aTSA and rTSA procedures performed by 3 surgeons using a single-platform shoulder system, with implant selection guided by glenoid morphology, rotator cuff status, and patient expectations.	aTSA and RTSA demonstrated similar survivorship at 2 and 8 yrs, with comparable complication rates, although aTSA patients achieved greater post-operative range of motion, indicating both implants provide reliable midterm outcomes and implant longevity.
Liu,^31^ 2022	Comparison of Reverse and Anatomic Total Shoulder Arthroplasty in Patients With an Intact Rotator Cuff and No Previous Surgery	International multi-institutional shoulder arthroplasty registry (single-platform system)	To compared clinical and radiographic outcomes of primary anatomic (aTSA) vs. reverse total shoulder arthroplasty (rTSA) in 370 matched patients with osteoarthritis, intact rotator cuffs, and no prior shoulder surgery, using a single implant platform and a minimum follow-up of 2 yrs. Outcomes were assessed with 6 functional scores and 4 range-of-motion measures.	At a mean follow-up of 41 mo, aTSA and rTSA demonstrated similar pain relief, function, and radiographic outcomes, with aTSA showing greater external rotation but a significantly higher complication rate. These results support rTSA as a safe and effective option in patients with intact rotator cuffs, offering comparable outcomes with lower perioperative morbidity.
Nazzal,^41^ 2023	Total shoulder arthroplasty vs. reverse total shoulder arthroplasty: Outcome comparison in osteoarthritis patients with or without concurrent rotator cuff deficiency	Nationwide Inpatient Sample (NIS) database	To compare outcomes of total shoulder arthroplasty (TSA) and reverse total shoulder arthroplasty (rTSA) in osteoarthritis patients with or without rotator cuff deficiency. Using the Nationwide Inpatient Sample (2011–2014), shoulder arthroplasties were identified, and patients were grouped by rotator cuff status and matched using propensity score analysis.	rTSA provided comparable complication rates to TSA in patients with rotator cuff deficiency but was associated with higher mechanical complications, post-operative pain, and costs in patients without rotator cuff damage, suggesting its use should be carefully considered in this population.
Trammell,^54^ 2023	Reverse shoulder arthroplasty with preservation of the rotator cuff for primary glenohumeral osteoarthritis has similar outcomes to anatomic total shoulder arthroplasty and reverse shoulder arthroplasty for cuff arthropathy	Single-institution clinical data/medical records	To compared outcomes of reverse total shoulder arthroplasty (rTSA) with an intact rotator cuff (+rcRSA) to rTSA for cuff arthropathy (-rcRSA) and anatomic TSA in patients with primary glenohumeral osteoarthritis, using pre-operative and post-operative range of motion, patient-reported outcomes (VAS, SSV, ASES), and complication rates from a single-institution cohort between 2015 and 2020 with minimum 12-mo follow-up.	At short-term follow-up, +rcRSA achieved similar pain relief, functional outcomes, and low complication rates compared with -rcRSA and TSA, although TSA demonstrated slightly superior internal and external rotation. Preservation of the rotator cuff in rTSA represents a viable option for patients with severe glenoid deformity or at risk for future cuff insufficiency.
Villacis,^56^ 2016	Clinical outcomes of anatomical vs. reverse total shoulder arthroplasty in patients with primary osteoarthritis, an intact rotator cuff, and limited forward elevation	Single-institution prospectively collected shoulder arthroplasty database	To compared clinical outcomes of aTSA vs. rTSA in patients with glenohumeral osteoarthritis, intact rotator cuffs, and limited forward elevation (FE ≤ 105°), using matched cohorts from a prospectively collected single-institution database and evaluating post-operative ROM, outcome scores, complications, and revision rates.	In patients with limited pre-operative FE, rTSA provided superior post-operative overhead ROM, abduction, and functional outcome scores, with similar rotational ROM, and demonstrated lower revision rates compared with aTSA.
Ponce,^46^ 2015	Complication rate and implant survival for reverse shoulder arthroplasty vs. total shoulder arthroplasty: results during the initial 2 years	California Office of Statewide Health Planning and Development (OSHPD) State Discharge Database	This study used a statewide database to compare complication and revision rates between reverse total shoulder arthroplasty (rTSA) and anatomic TSA from 2011–2013. Complications were assessed at 30 d, 90 d, 1 yr, and 2 yrs, with logistic regression identifying risk factors and Kaplan-Meier analysis evaluating implant survival.	rTSA was associated with significantly higher early and midterm complication rates, including infection and dislocation, though implant failure rates equalized by 1–2 yrs. Risk factors included male sex, younger age, pre-operative anemia, and workers' compensation status.
Mahoney,^34^ 2014	Comparative analysis of anatomic and reverse total shoulder arthroplasty: in-hospital outcomes and costs	Nationwide Inpatient Sample (NIS)	To compare perioperative outcomes between anatomic and reverse total shoulder arthroplasty using the 2011 Nationwide Inpatient Sample. Patients were analyzed for in-hospital complications, mortality, length of stay, discharge disposition, and hospital charges, with bivariate and multivariable logistic regression used to adjust for confounders.	Reverse total shoulder arthroplasty was independently associated with higher inpatient morbidity, mortality, prolonged hospital stay, non-routine discharge, and increased hospital costs compared with anatomic arthroplasty. These findings suggest rTSA should be offered judiciously and performed by experienced shoulder specialists.
Burnett,^8^ 2022	Readmission after shoulder arthroplasty	Single-institution clinical/hospital records	This retrospective study (2005-2011) evaluated shoulder arthroplasty procedures to determine 30-d, 60-d, and 90-d readmission rates and the incidence of CMS-defined “never events” across hemiarthroplasty (HA), total shoulder arthroplasty (TSA), and reverse total shoulder arthroplasty (rTSA).	Overall 90-d readmission occurred in 5.9% of patients, with a 1% incidence of never events. Re-admissions were more common within 30 d for HA and rTSA, highlighting the importance of early post-operative monitoring as reimbursement and quality metrics increasingly focus on short-term outcomes.
Valsamis,^55^ 2024	Characteristics and risk factors for 90-day readmission following shoulder arthroplasty	Electronic medical records from 2 tertiary referral health systems	This study analyzed primary shoulder arthroplasties across 2 tertiary health systems to identify patient and surgical characteristics associated with unplanned 90-d re-admissions. Univariable and multivariable logistic regression, including backward stepwise elimination, was used to select key predictors, and readmissions were classified as surgical or medical. Secondary outcomes included length of stay and discharge destination.	Reverse TSA, revision or fracture surgery, right-sided procedures, and other patient comorbidities were independently associated with higher 90-d re-admission risk, while short inpatient stay was not. Discharge to post-acute care facilities increased re-admission probability, highlighting the need for targeted pre-operative risk stratification and perioperative planning to reduce early complications and optimize outcomes.
Valsamis^55^	Reverse total shoulder replacement vs. anatomical total shoulder replacement for osteoarthritis: population based cohort study using data from the National Joint Registry and Hospital Episode Statistics for England	National Joint Registry linked to Hospital Episode Statistics (UK)	This registry-based cohort study compared reverse (rTSA) vs. anatomical (TSA) shoulder replacement in adults ≥60 with osteoarthritis and intact rotator cuffs, using propensity score matching and weighting to assess revision, 90-d serious adverse events, reoperations, hospital stay, function, and lifetime costs.	rTSA had lower short-term revision and reoperation risk, with no significant differences in long-term revision, complications, function, or costs, supporting its use as a safe alternative to TSA.

*SD*, standard deviation; *IQR*, interquartile range; *TSA*, total shoulder arthroplasty; *ASC*, ambulatory surgical center; *HSC*, hospital-based surgical center; *BMI*, body mass index; *rTSA*, reverse total shoulder arthroplasty; *VTE*, venous thromboembolism; *NRD*, National Readmissions Database; *aTSA*, anatomic total shoulder arthroplasty; *PACU*, post-anesthesia care unit; *ASA*, American Society of Anesthesiologists; *HA*, hemiarthroplasty; *GHOA*, glenohumeral osteoarthritis; *LOS*, length of stay; *BMI*, body mass index; *ED*, emergency department; *ER*, emergency room; *ANOVA*, analysis of variance; *OA*, osteoarthritis; *RCT*, randomized controlled trial; *PJI*, periprosthetic joint infection; *ROM*, range of motion; *VAS*, visual analog scale; *COPD*, chronic obstructive pulmonary disease; *SSV*, Subjective Shoulder Value; *2-ASC,* 2-Ambulatory Surgery Center (refers to a review/study conducted across two ambulatory surgery centers); *ACS-NSQIP,* American College of Surgeons National Surgical Quality Improvement Program; *ASES*, American Shoulder and Elbow Surgeons (score); *AUPRC,* area under the precision-recall curve; *AUROC,* area under the receiver operating characteristic curve; *CMS,* Centers for Medicare & Medicaid Services; *mFI-5,* 5-item modified Frailty Index; *SHAP,* shapley additive explanations; *DB,* database; *SNF,* skilled nursing facility; *+rcRSA,* reverse shoulder arthroplasty with preservation of the rotator cuff; *-rcRSA,* reverse shoulder arthroplasty for cuff arthropathy.

The majority of studies were conducted in the United States (n = 39), with a small number from France (n = 1), Italy (n = 1), the United Kingdom (n = 1), and multinational datasets including the United States and Europe (n = 4). Most studies were retrospective (n = 38), whereas a few were prospective (n = 5) or randomized controlled trials (n = 1). Many retrospective studies were cohort or database analyses, with some employing multicenter registries or propensity score methods to control for confounding factors (Table I).

### Risk of bias

Among the non-randomized studies assessed using the ROBINS-I tool, risk of bias varied across domains. Bias due to confounding was low in 8.9% of studies, moderate in 55.6%, and serious in 35.6%. Bias in the selection of participants was predominantly low risk (55.6%), with 42.2% at moderate risk and 2.2% at serious risk. Classification of interventions was largely low risk (93.3%), with only small proportions rated as moderate (4.4%) or serious (2.2%). Bias due to deviations from intended interventions was low in 91.1% and moderate in 8.9%, with no studies judged at serious risk. Bias due to missing data was low in 57.8%, moderate in 40.0%, and serious in 2.2% of studies. Measurement of outcomes was low risk in 71.1%, moderate in 26.7%, and serious in 2.2%. Selection of the reported result was low in 53.3% and moderate in 46.7%, with no serious risk identified. Overall, 40.0% of studies were judged at serious risk of bias, 51.1% at moderate risk, and 8.9% at low risk.

The single randomized controlled trial, Schumaier et al, had low risk of bias for randomization, deviations from intended interventions, and outcome measurement, with some concerns for effect of assignment and selection of reported results.^47^

### Indications for surgery

The primary surgical indications in the included studies were glenohumeral osteoarthritis, rotator cuff tear arthropathy or severe cuff deficiency, post-traumatic arthritis or sequelae of proximal humerus fractures, inflammatory arthritis, and avascular necrosis. Indications varied between studies and may contribute to differences in observed adverse event rates, particularly among rTSA cohorts.

### Types of complications

aTSA medical complications most commonly reported included acute blood-loss anemia or transfusion (0.2%-9.8%), acute renal failure or acute kidney injury (0.1%-6.2%), acute respiratory failure or respiratory distress (0.2%-3.8%), pneumonia (0.2%-3.5%), urinary tract infection (0.4%-6.1%), pulmonary embolism (0.1%-1.3%), deep vein thrombosis (0.1%-1.5%), myocardial infarction (0.2%-2.0%), sepsis or septic shock (0.1%-4.8%), cerebrovascular accident or stroke (0%-2.4%), post-operative hemorrhage (0.1%-1.1%), and mortality (0%-0.5%). Implant-related complications following aTSA included mechanical loosening (0.02%-2.8%), dislocation or instability (0.6%-5.5%), periprosthetic joint infection (0.4%-3.7%), periprosthetic fracture (0.03%-1.5%), rotator cuff or subscapularis failure (0.5%-4.3%), hematoma or seroma formation (0%-1.0%), stiffness or arthrofibrosis (0%-3.0%), wound dehiscence or superficial wound complications (0.1%-2.2%), persistent post-operative pain (0.2%-3.1%), and revision or re-operation (0.2%-2.8%).

rTSA medical complications included acute blood-loss anemia or transfusion (0.4%-10.7%), acute renal failure or acute kidney injury (0.2%-7%), acute respiratory failure or respiratory distress (0.2%-3.8%), pneumonia (0.5%-6.2%), urinary tract infection (0.5%-11.0%), pulmonary embolism (0.1%-1.3%), deep vein thrombosis (0.2%-3.6%), myocardial infarction (0.2%-4.7%), sepsis or septic shock (0.3%-6.7%), cerebrovascular accident or stroke (0%-1.7%), post-operative hemorrhage (0.1%-1.2%), and mortality (0%-0.7%). Implant-related complications following rTSA included dislocation or instability (0.9%-8.5%), periprosthetic joint infection (0.9%-4.9%), mechanical loosening (0.02%-2.8%), periprosthetic fracture (0.03%-2.5%), acromial or scapular spine fracture (0.9%-1.7%), hematoma or seroma formation (0.06%-1.3%), stiffness or arthrofibrosis (0%-1.0%), wound dehiscence or superficial wound complications (0%-2.2%), persistent post-operative pain (0.2%-1.2%), and revision or re-operation (0.3%-3.9%).

### Comparative meta-analysis

#### Serious adverse events

For SAEs, 26 studies including 553,078 rTSA patients and 454,144 aTSA patients were eligible for comparative meta-analysis (Fig. 4 and Table 3).^2^, ^3^, ^4^, ^5^^,^^8^, ^9^, ^10^^,^^14^^,^^16^^,^^20^^,^^22^^,^^26^^,^^29^^,^^31^^,^^33^^,^^35^^,^^37^^,^^40^^,^^41^^,^^44^^,^^46^^,^^49^^,^^53^, ^54^, ^55^, ^56^ rTSA was associated with significantly higher odds of SAE compared with aTSA (OR: 1.38; 95% CI: 1.10-1.74; *P* = .006), corresponding to a 38% increase in odds. Substantial heterogeneity was observed (I^2^ = 98.5%; τ^2^ = 0.27; *P* < .001) (Supplementary Fig. S1).

**Figure 4:**
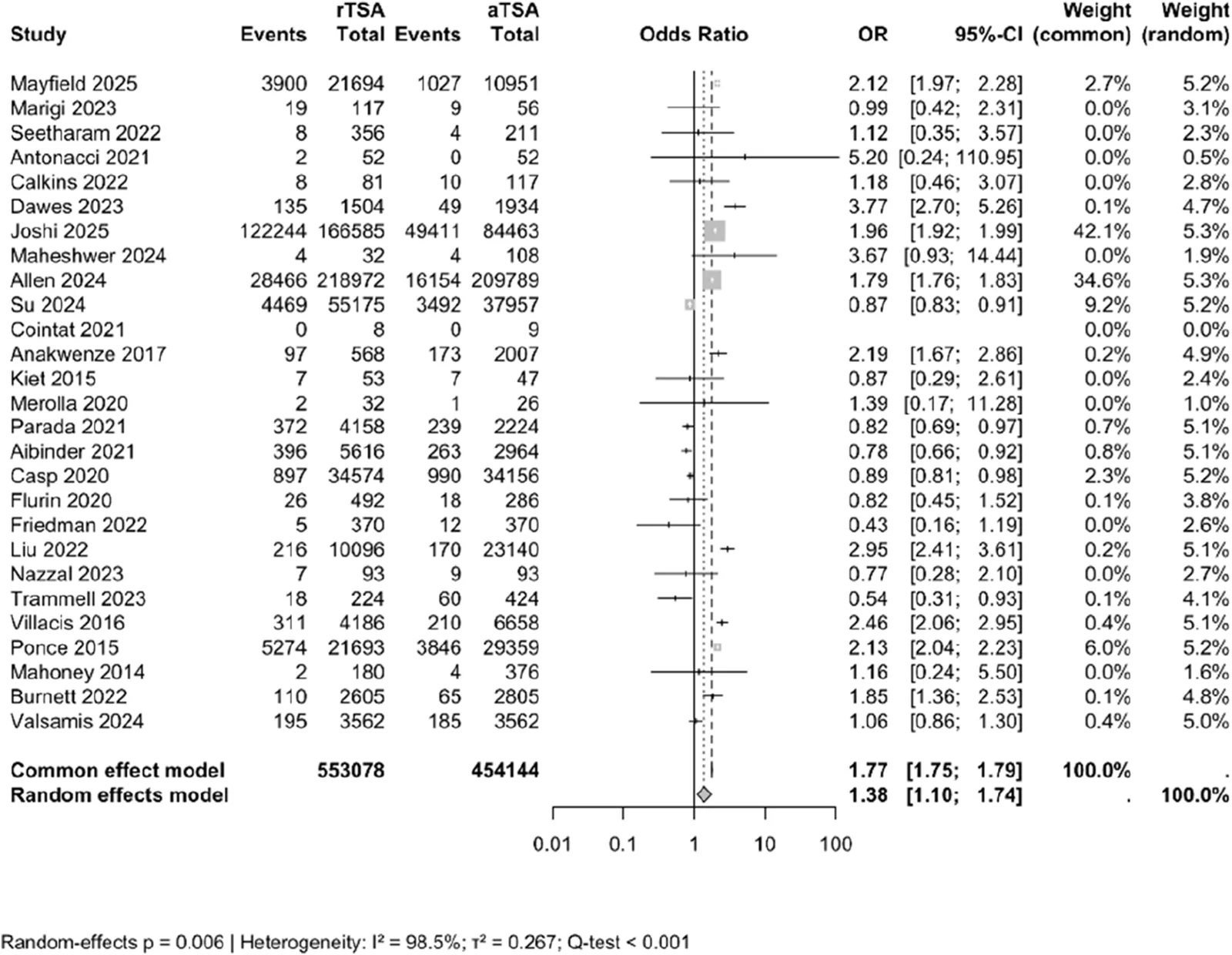
Comparative meta-analysis aTSA vs. rTSA SAE. *OR*, odds ratio; *CI*, confidence interval; *aTSA*, anatomic total shoulder arthroplasty; *rTSA*, reverse total shoulder arthroplasty; *SAE*, serious adverse event.

**Table III:** Comparative and pooled single-arm analyses of serious adverse events and 30-day and 90-day re-admission rates following anatomic vs. reverse total shoulder arthroplasty.

Outcome	Comparative studies, n	Comparative patients, n	Comparative OR (95% CI)	*P* value	I^2^	τ^2^	aTSA single-arm studies, n	aTSA patients, n	aTSA pooled rate (95% CI)	I^2^	τ^2^	rTSA single-arm studies, n	rTSA patients, n	rTSA pooled rate (95% CI)	I^2^	τ^2^
Serious adverse events	26	1,007,222	1.38 (1.10-1.74)	.006	98.5%	0.27	29	530,339	5.28% (3.48-7.92)	100%	1.284	41	884,528	8.78% (5.70-12.00)	>99.9%	1.657
30-d re-admission	5	268,866	1.71 (1.47-1.98)	<.001	31.5%	0.01	9	141,911	1.24% (0.66-2.31)	98.7%	0.73	13	191,855	3.16% (2.22-4.49)	95.9%	0.32
90-d re-admission	12	367,388	1.53 (1.34-1.75)	<.001	64.2%	0.02	16	219,430	3.05% (2.01-4.60)	99.6%	0.57	24	544,785	4.09% (2.87-5.79)	99.7%	0.68

*OR*, odds ratio; *CI*, confidence interval; *I*^*2*^, percentage of variability across studies attributable to between-study heterogeneity; *τ*^*2*^, between-study variance; *aTSA*, anatomic total shoulder arthroplasty; *rTSA*, reverse total shoulder arthroplasty.

#### 30-day re-admission rate

Thirty-day re-admission rate was evaluated in 5 studies including 173,013 rTSA patients and 95,853 aTSA patients (Fig. 5).^16^^,^^26^^,^^34^^,^^52^^,^^56^ rTSA demonstrated significantly higher odds of 30-day re-admission compared with aTSA (OR: 1.71; 95% CI: 1.47-1.98; *P* < .001). There was no statistically significant evidence of between-study heterogeneity (I^2^ = 31.5%; τ^2^ = 0.01; *P* = .212) (Supplementary Fig. S2).

**Figure 5:**
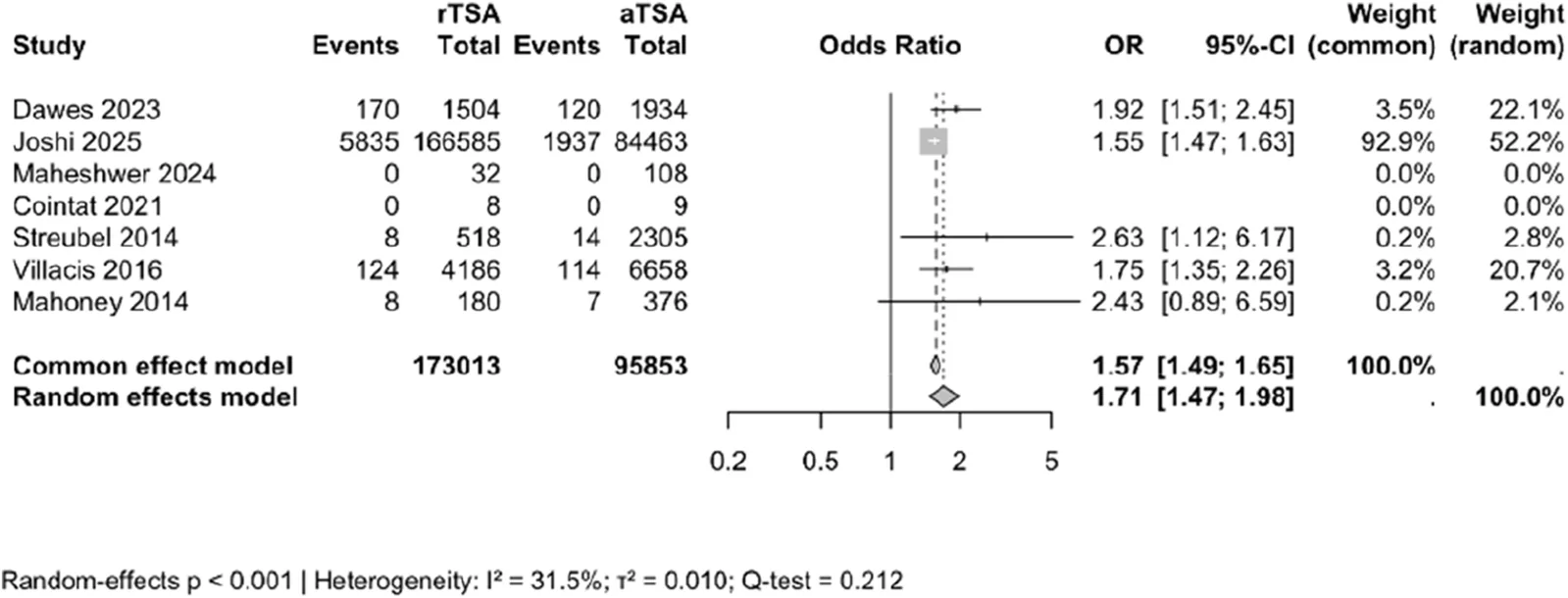
Comparative meta-analysis aTSA vs. rTSA 30-day readmission.*OR*, odds ratio; *CI*, confidence interval; *aTSA*, anatomic total shoulder arthroplasty; *rTSA*, reverse total shoulder arthroplasty.

#### 90-day re-admission rate

Ninety-day re-admission rate was assessed in 12 studies including 230,141 rTSA patients and 137,247 aTSA patients (Fig. 6).^4^^,^^5^^,^^9^^,^^16^^,^^26^^,^^34^^,^^35^^,^^49^^,^^52^^,^^53^^,^^56^^,^^58^ rTSA was associated with significantly increased odds of 90-day re-admission compared with aTSA (OR: 1.53; 95% CI: 1.34-1.75; *P* < .001). Moderate heterogeneity was present (I^2^ = 64.2%; τ^2^ = 0.02; *P* = .001) (Supplementary Fig. S3).

**Figure 6:**
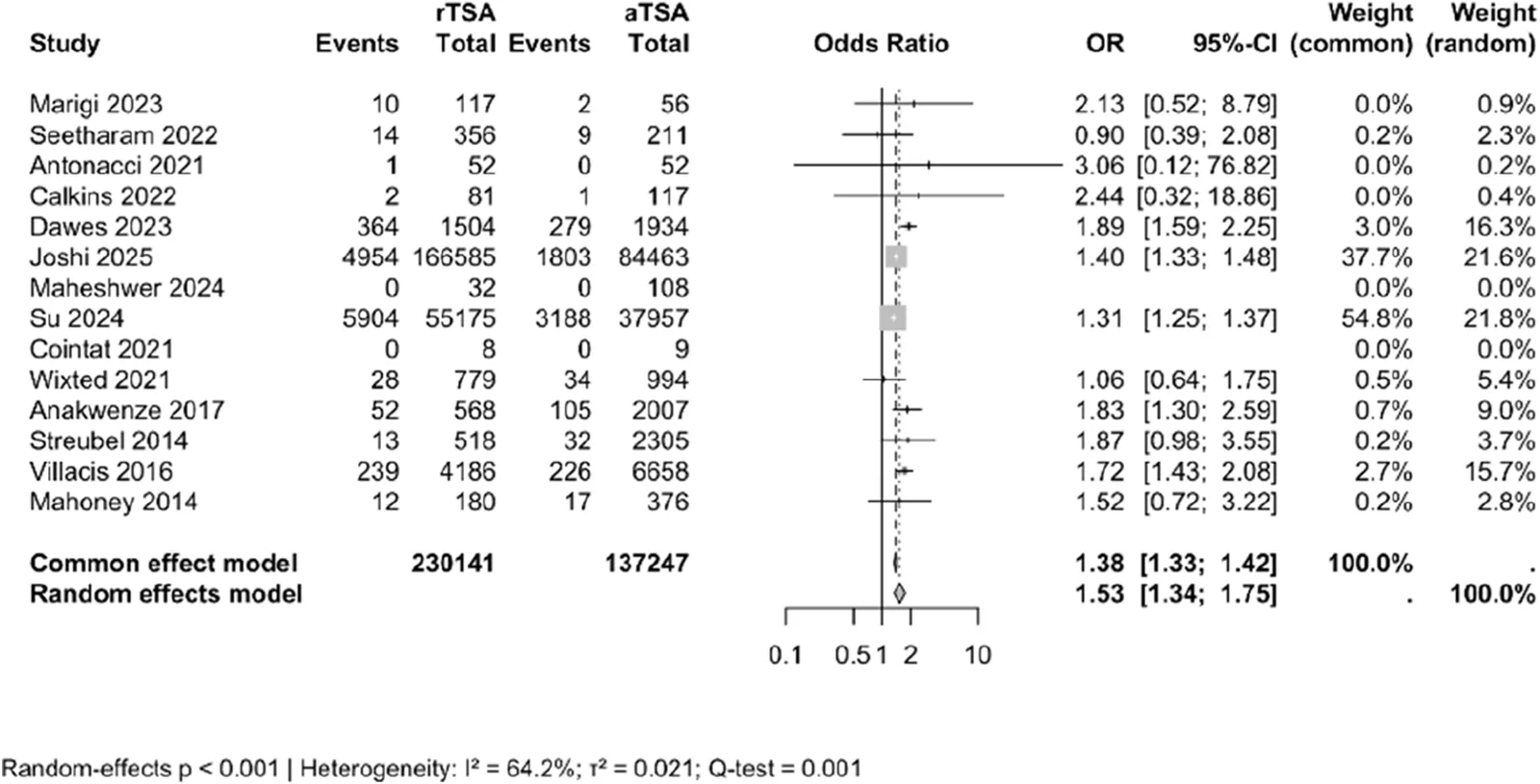
Comparative meta-analysis aTSA vs. rTSA 90-day readmission. *OR*, odds ratio; *CI*, confidence interval; *aTSA*, anatomic total shoulder arthroplasty; *rTSA*, reverse total shoulder arthroplasty.

### Single-arm meta-analysis

In the single-arm meta-analysis for aTSA, the pooled SAE rate was 5.28% (95% CI: 3.48-7.92; I^2^ = 100%; τ^2^ = 1.284) across 29 studies including 530,339 patients, while rTSA demonstrated a higher pooled SAE rate of 8.78% (95% CI: 5.7-12; I^2^ > 99.9%; τ^2^ = 1.657) across 41 studies including 884,528 patients (Figs. 7 and 8).^2^, ^3^, ^4^, ^5^, ^6^^,^^8^, ^9^, ^10^^,^^12^^,^^15^, ^16^, ^17^, ^18^^,^^20^, ^21^, ^22^, ^23^, ^24^, ^25^, ^26^^,^^28^, ^29^, ^30^, ^31^, ^32^, ^33^, ^34^, ^35^, ^36^, ^37^^,^^39^, ^40^, ^41^, ^42^^,^^44^^,^^46^^,^^47^^,^^49^^,^^53^, ^54^, ^55^, ^56^

**Figure 7:**
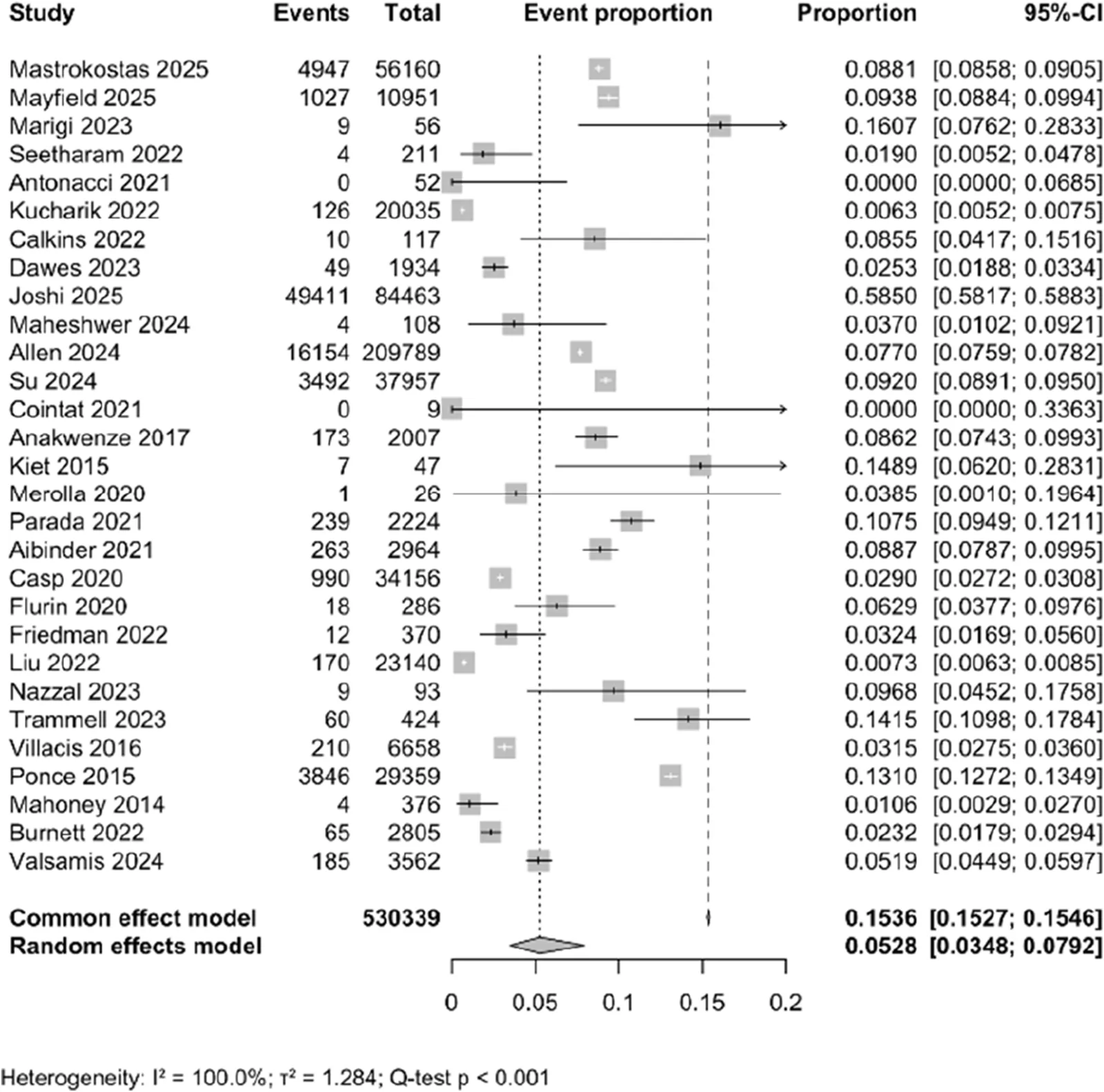
Single-arm meta-analysis aTSA SAE. *CI*, confidence interval; *aTSA*, anatomic total shoulder arthroplasty; *SAE*, serious adverse event.

**Figure 8:**
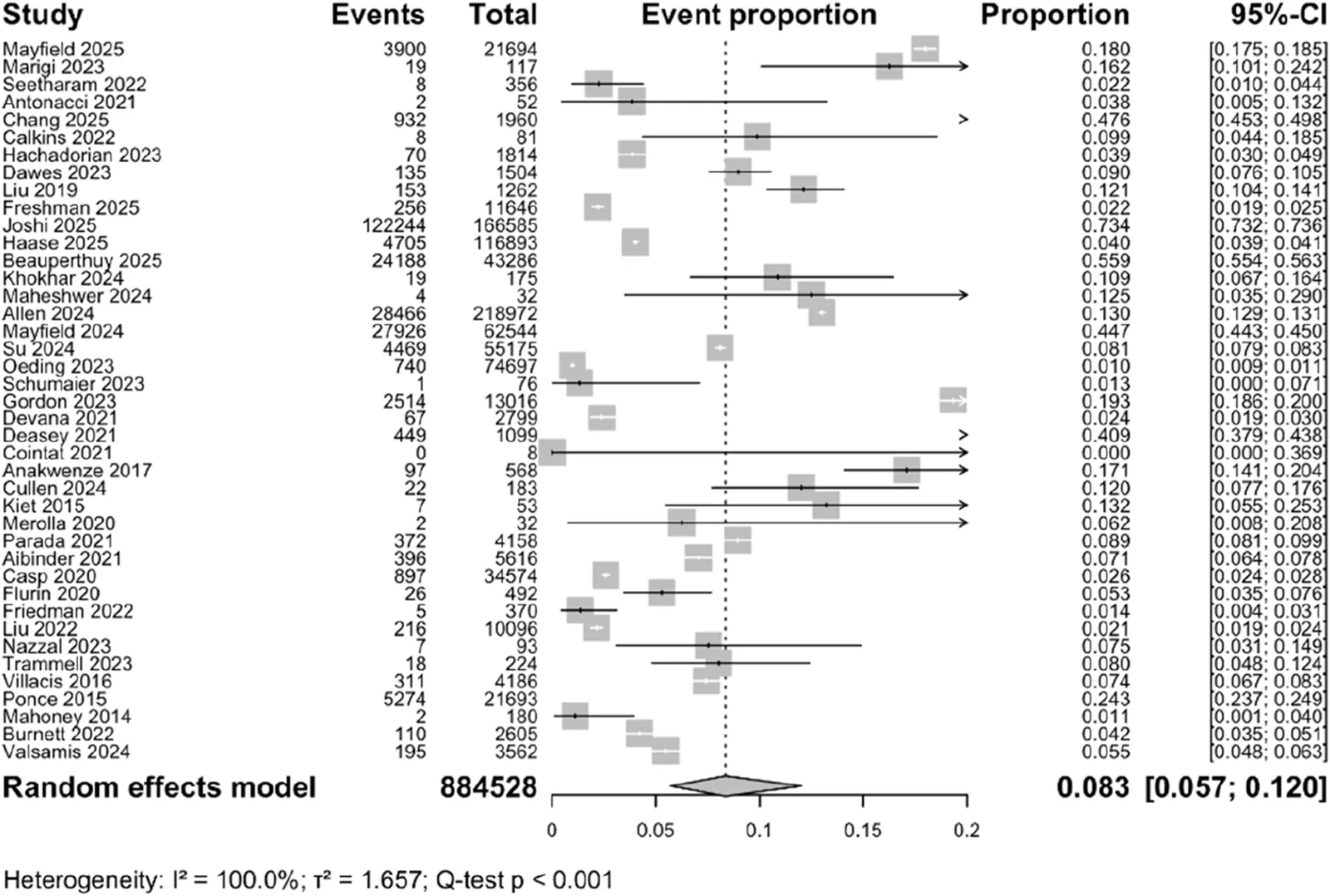
Single-arm meta-analysis rTSA SAE. *CI*, confidence interval; *rTSA*, reverse total shoulder arthroplasty; *SAE*, serious adverse event.

For 30-day re-admission rate was lowest following aTSA, with a pooled rate 1.241% (95% CI: 0.664-2.308; I^2^ = 98.7%; τ^2^ = 0.73) across 9 studies including 141,911 patients, compared with 3.164% (95% CI: 2.22-4.49; I^2^ = 95.9%; τ^2^ = 0.32) for rTSA across 13 studies including 191,855 patients (Figs. 9 and 10).^12^, ^13^, ^14^^,^^16^, ^17^, ^18^^,^^21^^,^^26^^,^^30^^,^^33^^,^^34^^,^^39^^,^^47^^,^^52^^,^^56^

**Figure 9:**
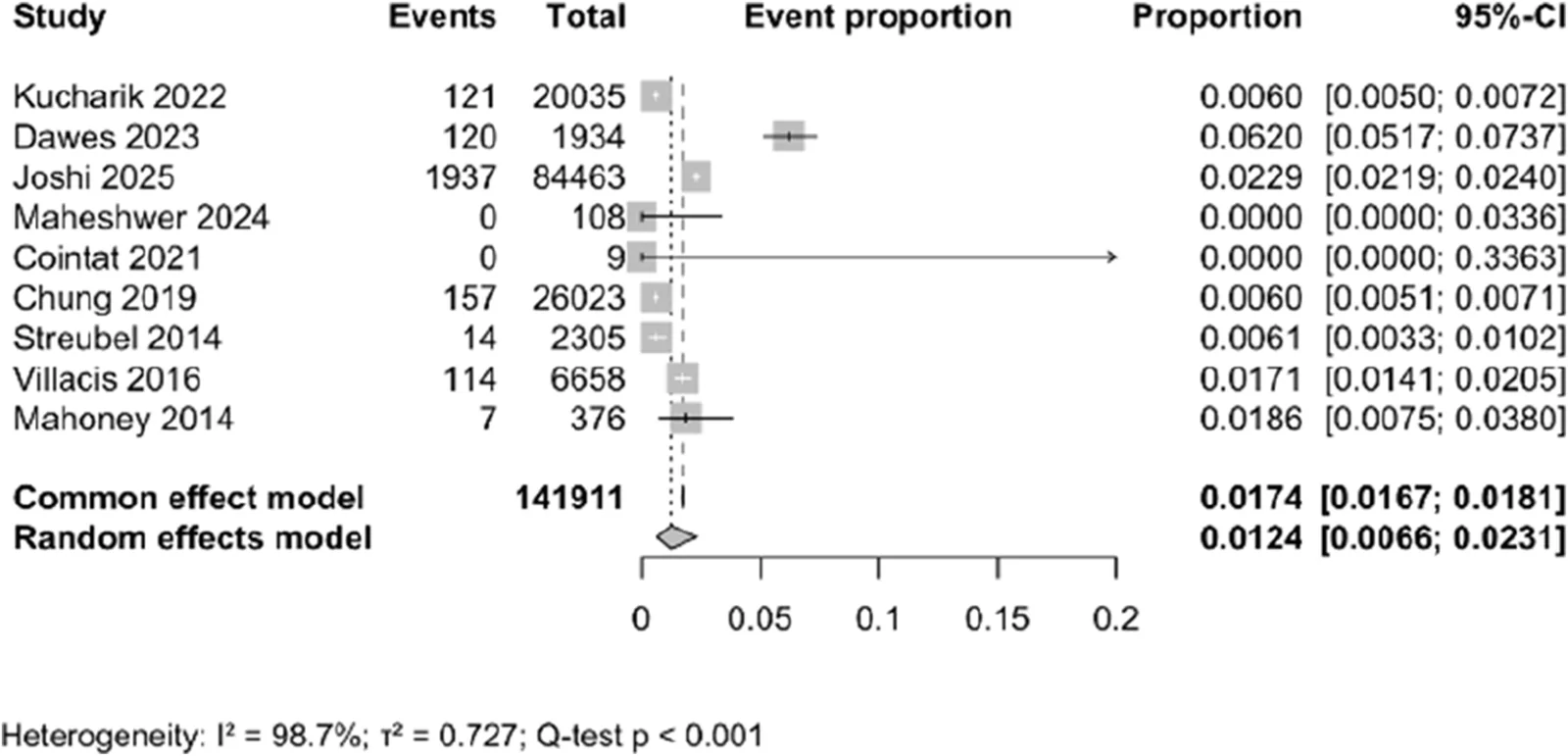
Single-arm meta-analysis aTSA 30-day readmission. *CI*, confidence interval; *aTSA*, anatomic total shoulder arthroplasty.

**Figure 10:**
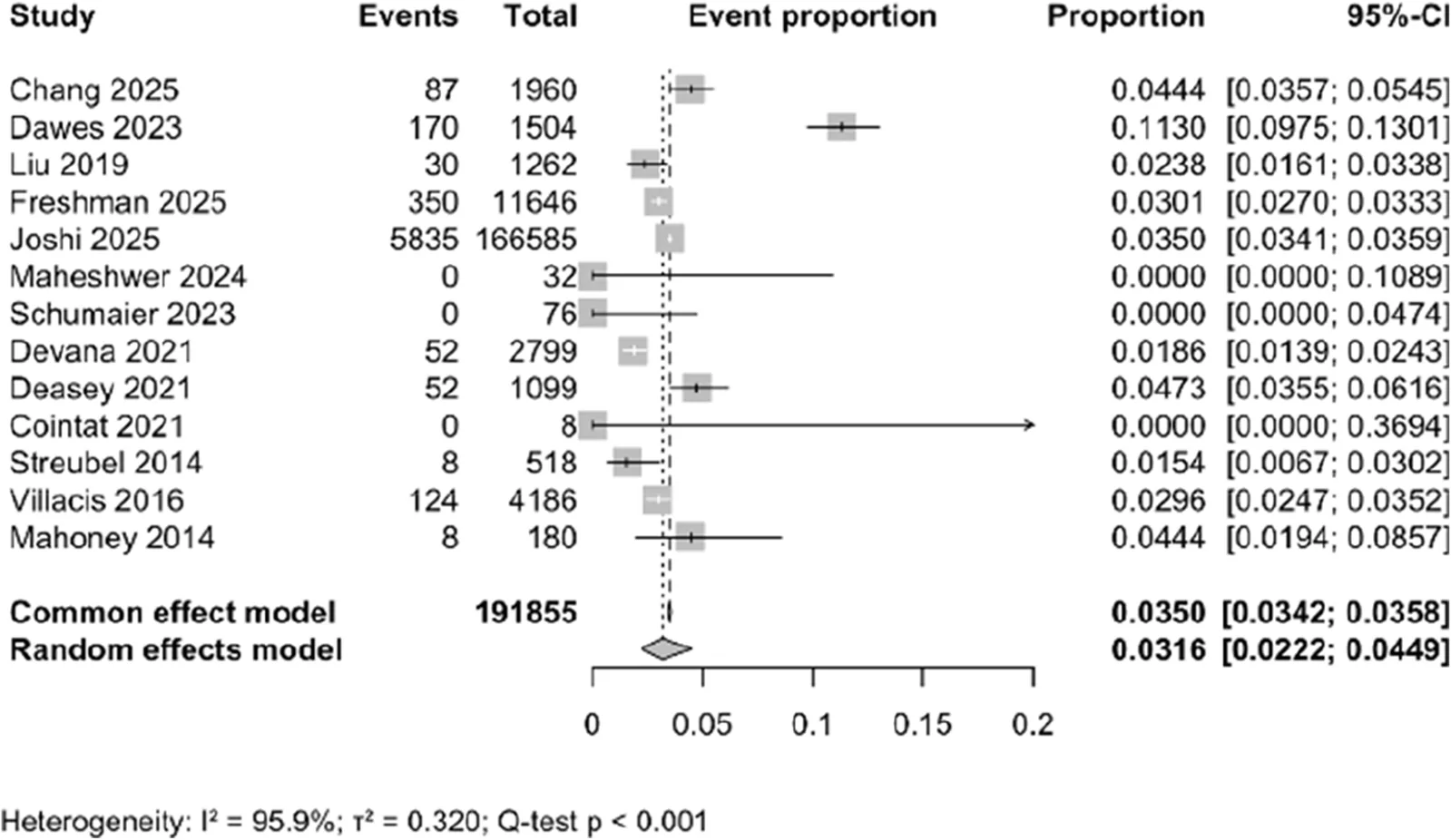
Single-arm meta-analysis rTSA 30-day readmission. *CI*, confidence interval; *rTSA*, reverse total shoulder arthroplasty.

A similar pattern was observed for 90-day re-admission rate for aTSA, the pooled rate was 3.05% (95% CI: 2.01-4.60; I^2^ = 99.6%; τ^2^ = 0.57) across 16 studies including 219,430 patients, while rTSA demonstrated a pooled 90-day re-admission rate of 4.09% (95% CI: 2.87-5.79; I^2^ = 99.7%; τ^2^ = 0.68) across 24 studies including 544,785 patients (Figs. 11 and 12).^4^, ^5^, ^6^^,^^9^^,^^12^, ^13^, ^14^, ^15^, ^16^^,^^23^, ^24^, ^25^, ^26^^,^^28^^,^^33^, ^34^, ^35^, ^36^^,^^39^^,^^42^^,^^47^^,^^49^^,^^52^^,^^53^^,^^56^^,^^58^

**Figure 11:**
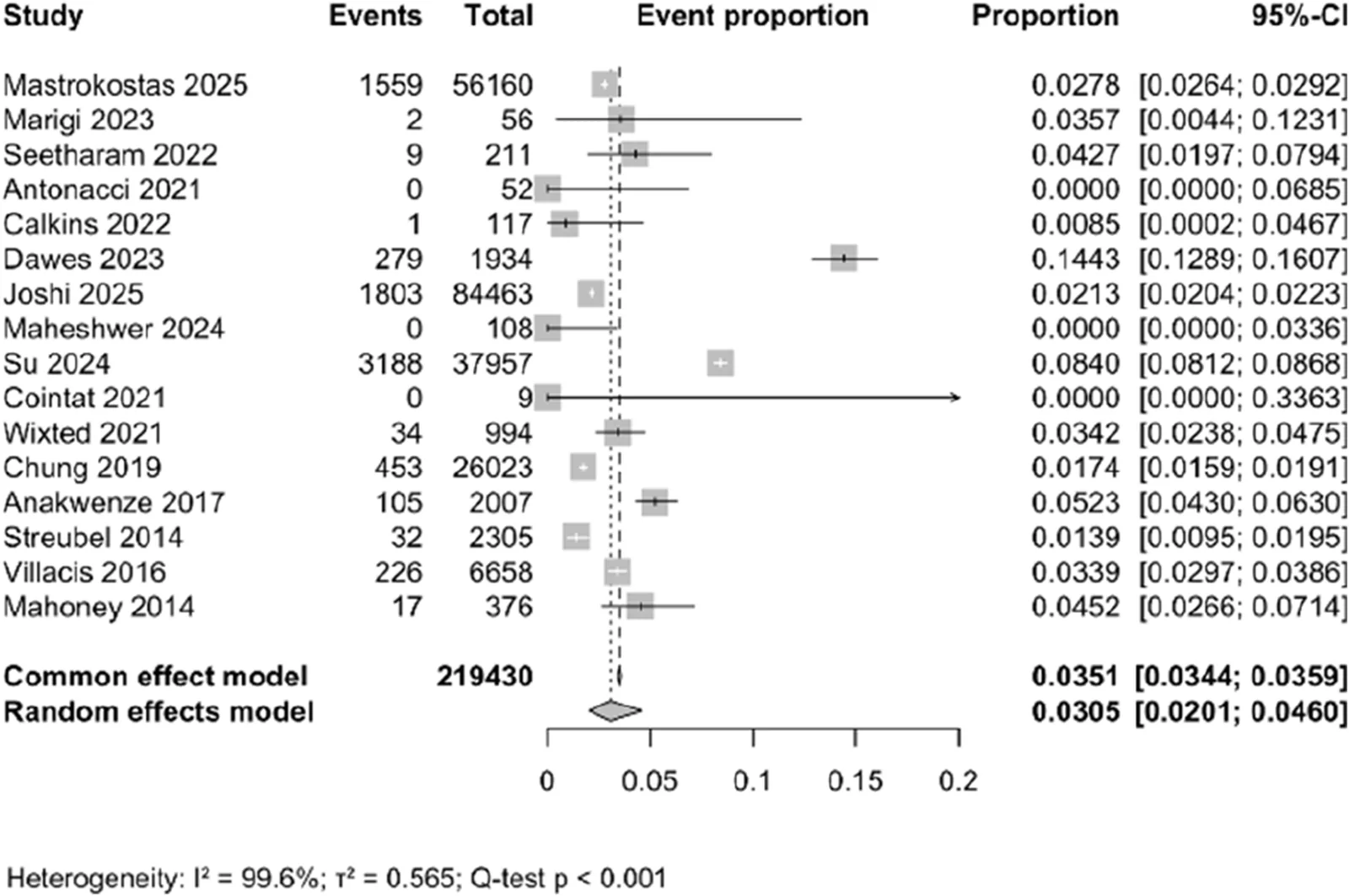
Single-arm meta-analysis aTSA 90-day readmission. *CI*, confidence interval; *aTSA*, anatomic total shoulder arthroplasty.

**Figure 12:**
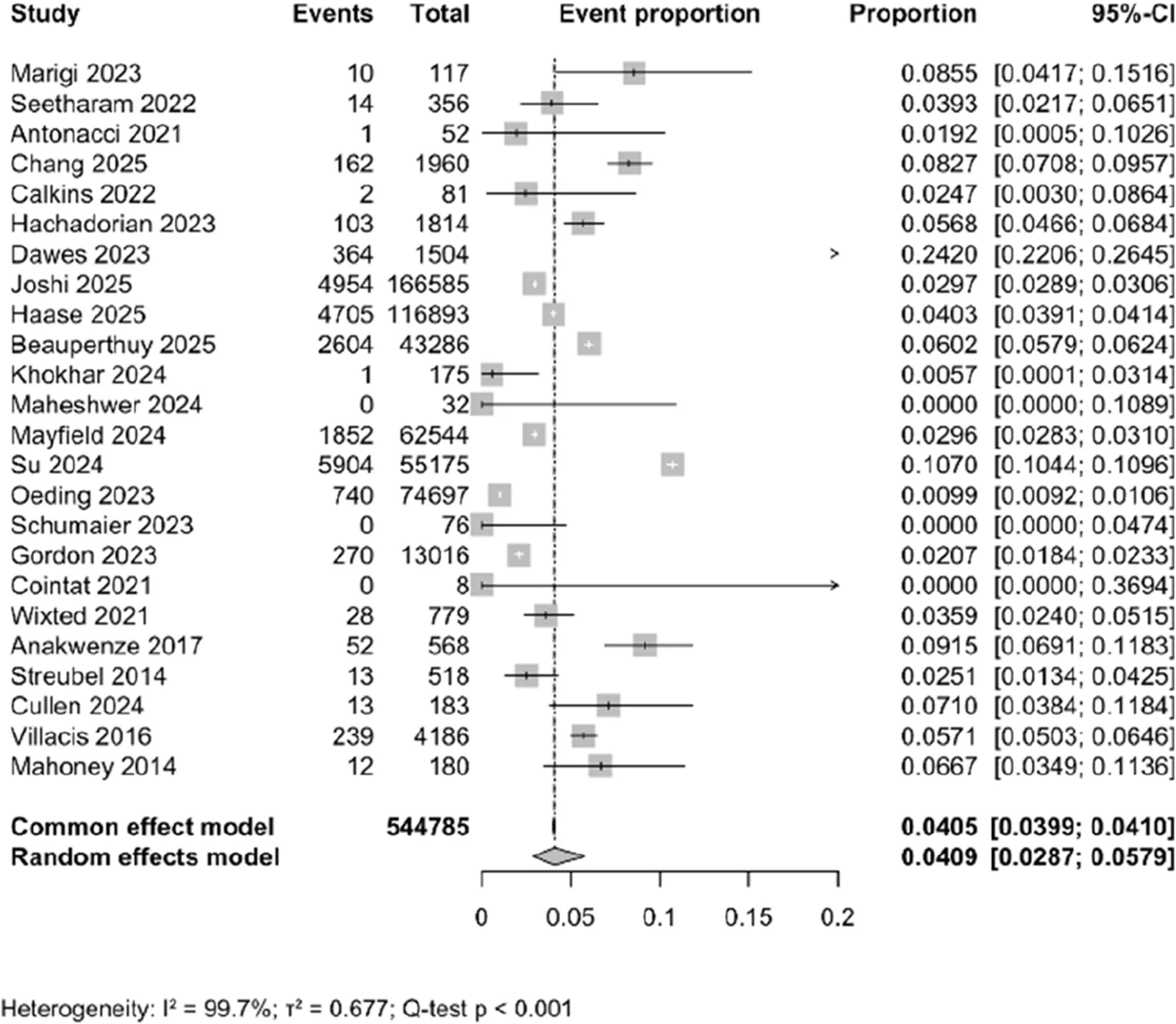
Single-arm meta-analysis rTSA 90-day readmission. *CI*, confidence interval; *rTSA*, reverse total shoulder arthroplasty.

## Discussion

### Principal findings

This review of 46 studies including more than 1.4 million patients demonstrates that rTSA is associated with higher perioperative morbidity and re-admission compared with aTSA. Comparative meta-analyses revealed that rTSA carried 38% increased odds of SAEs, 71% higher odds of 30-day re-admission, and 53% higher odds of 90-day re-admission. Single-arm analyses confirmed higher pooled SAE rates for rTSA (8.78%) vs. aTSA (5.28%) and greater 30-day and 90-day re-admission rates (3.16% vs. 1.24% and 4.09% vs. 3.05%, respectively). Although both procedures exhibited low absolute rates of mortality and major medical complications, rTSA demonstrated increased rates of dislocation, periprosthetic joint infection, and revision, whereas aTSA was more frequently complicated by mechanical loosening and rotator cuff failure. These findings highlight the importance of careful pre-operative risk stratification and optimization of comorbidities in patients undergoing shoulder arthroplasty, particularly those at a higher baseline risk, rather than suggesting that selection of one arthroplasty type over another would independently reduce complications.

### Clinical significance

These findings should be interpreted in the context of the distinct patient populations undergoing rTSA and aTSA, which often differ in age, comorbidity burden, functional status, and surgical indication. The higher incidence of SAEs and 30-day and 90-day re-admissions following rTSA underscores the need for procedure-specific pre-operative planning, including optimization of comorbidities, careful implant selection, and targeted post-operative monitoring. Awareness of the differential complication profiles rTSA with a greater risk of dislocation, infection, and revision, and aTSA with higher rates of mechanical loosening and rotator cuff failure enables surgeons to tailor surgical technique and rehabilitation strategies to individual patient risk. To our knowledge, this is the first systematic review demonstrating statistically higher complication and early re-admission rates for rTSA; previous meta-analyses reported no significant differences.^1^^,^^19^ These findings provide population-level benchmarks that may support shared decision-making and perioperative risk stratification, while recognizing that the observed differences cannot be interpreted as causal effects of arthroplasty type. Patient counseling should therefore consider individual comorbidity burden, surgical indication, and baseline risk when discussing expected post-operative outcomes.

### Highlights from meta-analysis

#### Serious adverse events

rTSA consistently showed higher perioperative morbidity across population-level studies, although study-specific findings varied. Antonacci et al reported the highest SAE rates and widest variability for rTSA, reflecting a case-mix skewed toward older, comorbid patients and complex indications, including fractures, cuff tear arthropathy, and revision surgery.^5^ These factors, rather than implant design alone, likely drive the elevated complication rates observed. In contrast, aTSA was frequently performed in younger, healthier patients with primary osteoarthritis, often using structured outpatient pathways and same-day discharge protocols, resulting in substantially lower SAE rates.^5^

Variation in surgical indication is an important consideration when interpreting the higher adverse event rates observed following rTSA. Reverse arthroplasty is frequently performed for rotator cuff deficiency, fracture-related pathology, and more complex shoulder disease, populations that may have greater baseline frailty and comorbidity than patients undergoing aTSA for primary osteoarthritis. Therefore, differences in adverse event rates may partly reflect underlying patient selection and surgical indication rather than an independent effect of implant design.

Interestingly, Friedman et al observed lower complication rates for rTSA compared with aTSA in a cohort restricted to primary osteoarthritis, intact rotator cuffs, and no prior surgery.^22^ This demonstrates that when case-mix is controlled, rTSA can achieve safety profiles comparable to aTSA, highlighting the importance of patient selection and indication bias in interpreting comparative outcomes. Large registry studies, including Joshi et al and Allen et al, reinforced the overall pattern that rTSA was associated with increased early morbidity, particularly in elderly populations or patients with neurologic disorders.^3^^,^^26^ Allen et al reported that neurologic patients undergoing rTSA experienced higher 180-day complication rates (OR: 1.47; 95% CI: 1.29-1.67; *P* < .001), increased mortality (OR: 2.27; 95% CI: 1.25-4.14; *P* = .007), longer hospital stays, and higher rates of non-home discharge, illustrating the amplified risk in vulnerable populations.^3^

Overall elevated SAEs after rTSA are largely driven by patient factors and surgical complexity rather than implant choice alone, emphasizing the critical role of pre-operative risk stratification and optimization.

#### 30-day re-admission

Registry-based studies consistently show higher 30-day re-admissions after rTSA. Joshi et al reported 3.5% vs. 2.3% for aTSA in patients aged ≥70 years, with risk largely attributable to surgical complications such as dislocation, infection, mechanical loosening, and scapular fractures.^26^ Older age, female sex, comorbidities (chronic obstructive pulmonary disease, chronic kidney disease, diabetes, cardiovascular disease, anemia, and osteoporosis), and longer length of stay independently increased re-admission risk, underscoring the additive effect of patient frailty and surgical complexity.^26^

In contrast, smaller, outpatient cohorts showed minimal early re-admissions. Maheshwer et al observed zero 30-day re-admissions for aTSA, and only minor, re-admission events for rTSA, reflecting stringent inclusion criteria (age <70, body mass index <40, limited comorbidities) and structured perioperative care.^33^ Similarly, Cointat et al reported 0% 30-day re-admissions following outpatient aTSA in carefully selected American Society of Anesthesiologists I-II patients, highlighting the impact of structured patient selection and optimization of comorbidities on early post-operative outcomes.^14^ All patients received a structured perioperative care pathway, including general and locoregional anesthesia with a perineural interscalene catheter, followed by oral analgesics, and were only discharged if the Post-Anaesthetic Discharge Scoring System was >9/10 and pain was adequately controlled (visual analog scale <5/10).^14^ Rigorous pre-operative education, provided by the surgeon, anesthesiologist, and nursing coordinator, included instruction on the use of the shoulder sling and self-management of analgesia.^14^

These findings highlight that while rTSA carries higher early re-admission risk at the population level, careful patient selection and standardized perioperative pathways can mitigate this risk, emphasizing the importance of context-specific interpretation when counseling patients and planning shoulder arthroplasty.

#### 90-day re-admission

Patterns for 90-day re-admissions largely mirror those for 30 days, although study-specific variation exists. Seetharam et al uniquely observed lower 90-day re-admissions for rTSA than for aTSA, attributed to rigorous patient selection, increased outpatient procedures during the COVID-19 era, and standardized perioperative protocols, including regional anesthesia, multimodal analgesia, and early mobilization.^49^ Wixted et al reported the most conservative estimate, with an OR of 1.06 (95% CI: 0.64-1.75) for rTSA vs. aTSA. Low overall event rates, high procedural standardization, and management in a high-volume center likely mitigated the impact of intraoperative fractures on short-term outcomes.^58^

Although rTSA generally confers higher 90-day re-admission risk, outcomes are highly dependent on institutional protocols, patient selection, and perioperative optimization. Exceptional perioperative pathways can reduce or even invert the expected risk, as shown in select high-quality cohorts.

#### Comparison with current literature

Prior studies have delineated the relative safety and effectiveness of aTSA vs. rTSA. Dragonas et al in their systematic review included 2,731 shoulders in patients >70 years with primary osteoarthritis and intact rotator cuffs (1,472 aTSAs and 1,259 rTSAs).^19^ rTSA had a significantly lower revision rate than aTSA (OR: 0.50; 95% CI: 0.30-0.84; *P* = .009), while overall complication rates were comparable (7.9% vs. 8.8%; OR: 0.98; 95% CI: 0.34-2.86; *P* = .97).^19^ Functional outcomes favored aTSA, with higher post-operative Constant Murley scores (80 vs. 68; *P* < .001).^19^ Similarly, Kent et al evaluated 79,037 rTSA procedures and reported a 90-day complication rate of 5%, revision rate of 1.5%, and 90-day mortality of 1.1%, confirming that early adverse events remain uncommon despite patient age and comorbidity.^27^

Large institutional cohorts clarify early re-admission and complication risk after TSA. Burnett et al analyzed 5,410 TSAs (2,805 aTSAs and 2,605 rTSAs), reporting a 3.2% 90-day re-admission rate, predominantly surgical (63%; *P* < .001).^8^ Key multivariable predictors included rTSA (OR: 2.5; 95% CI: 1.9-3.3), revision surgery (OR: 3.2; 95% CI: 2.1-4.8), fracture indication (OR: 2.8; 95% CI: 1.9-4.2), and American Society of Anesthesiologists ≥3 (OR: 1.6; 95% CI: 1.2-2.2), while short stays (≤1 day) were not associated with increased likelihood of re-admission (*P* = .684) with the predictive model demonstrating moderate discrimination, with an area under the curve of 0.716-0.724.^8^ These data support that TSA is safe in carefully selected patients, with risk stratification based on procedure type and comorbidities critical to optimize early outcomes and resource use.

Notably, our meta-analysis differs from previous studies by demonstrating statistically significant differences in both SAEs and early re-admissions between rTSA and aTSA. While prior reviews and smaller cohort studies often reported comparable complication rates or non-significant differences, our pooled analysis of more than 1.4 million patients provides sufficient power to detect these differences. Specifically, rTSA was associated with higher odds of SAEs (OR: 1.38), 30-day re-admission (OR: 1.71), and 90-day re-admission (OR: 1.53), providing robust, population-level benchmarks that extend existing literature. These findings underscore that, despite low absolute event rates, rTSA carries a consistently higher early risk than aTSA, highlighting the importance of procedure-specific risk stratification, patient counseling, and perioperative optimization.

### Limitations

This review is primarily limited by the predominance of retrospective cohort and registry-based studies, which are inherently susceptible to residual confounding, coding errors, and variable outcome definitions. Many studies carried moderate to serious risk of bias, particularly due to confounding. This is important because the choice between aTSA and rTSA is influenced by factors such as age, comorbidity burden, surgical indication, and rotator cuff status, which may not be fully captured or adjusted for in retrospective and administrative datasets. Therefore, observed differences in adverse events and re-admissions may partly reflect differences in baseline patient characteristics rather than the effect of arthroplasty type itself. Considerable heterogeneity across studies likely reflects differences in patient demographics, surgical indications, perioperative care, and institutional protocols rather than methodological flaws alone, so pooled estimates should be interpreted as population-level benchmarks. Key variables such as frailty, surgeon volume, implant design, and standardized definitions of SAEs were inconsistently reported. Furthermore, while single-arm meta-analyses have provided valuable estimates of complication and re-admission rates for aTSA and rTSA, they are inherently limited by the absence of direct comparators, which restricts causal inference. Also, the predominance of US-based data may limit generalizability to other healthcare systems and practice settings.

Potential population overlap between included studies represents an additional limitation, particularly among studies using large administrative databases, registries, or institutional cohorts. Some studies may have included overlapping patient populations or study periods, resulting in non-independence of observations and potential inflation of the apparent sample size and precision of pooled estimates. Because individual patient-level data were unavailable, the extent of any overlap could not be reliably quantified. The aggregate sample size should, therefore, be interpreted cautiously and should not be assumed to represent an equivalent number of independent patients.

### Future directions

Future studies should prioritize prospective data collection with harmonized definitions of early adverse events and re-admissions. Incorporation of patient-level factors such as frailty, functional status, and social determinants of health may improve early risk stratification, particularly for rTSA. Comparative analyses adjusting for surgical indication and comorbidity burden are needed to distinguish procedure-specific risk from patient selection effects, and linkage of early outcomes to longer-term revision, functional, and cost-related outcomes would enhance clinical relevance.

## Conclusion

In this review of more than 1.4 million patients, rTSA was associated with higher odds of SAEs and 30-day and 90-day re-admissions compared with aTSA, although absolute complication rates remained low for both procedures. These findings highlight the need for procedure-specific risk stratification, optimization of comorbidities, and enhanced perioperative monitoring in patients undergoing rTSA, particularly older and medically complex individuals. Improved patient selection and targeted perioperative pathways may reduce early complications and re-admissions, supporting safer and more value-based shoulder arthroplasty care.

## Disclaimers:

Funding: No funding was disclosed by the authors.

Conflicts of interest: The authors, their immediate families, and any research foundations with which they are affiliated have not received any financial payments or other benefits from any commercial entity related to the subject of this article.
