# Supplementary material for: Serious adverse events and 30-day and 90-day re-admission rates following anatomic versus reverse total shoulder arthroplasty: a systematic review and meta-analysis
Source: JSES Rev Rep Tech. 2026 Sep 4;7(1):100875. doi: 10.1016/j.xrrt.2026.100875 (PMC13755963; doi:10.1016/j.xrrt.2026.100875)
Supplement: Supplementary file 2 [file mmc2.docx]

Search String

(shoulder arthroplasty OR total shoulder replacement OR total shoulder arthroplasty) AND (adverse events OR serious adverse events OR AE OR complications OR Complication OR mortality OR death OR VTE OR "embolism" or "Venous Thromboembolism" OR MI OR "myocardial infarction" OR Stroke OR PE OR "Pulmonary Embolism" OR DVT OR "Deep Vein Thrombosis" OR sepsis OR infection OR PJI OR Revision OR Reoperation OR Dislocation OR Neurovascular OR ICU OR ITU OR "Intensive Care") AND (readmission OR (readmission AND (30 AND Day OR 30d OR 30-d OR 30-day)) OR hospital readmission)

Supplementary Table 1 – Search String
