# Supplementary material for: Serious adverse events and 30-day and 90-day re-admission rates following anatomic versus reverse total shoulder arthroplasty: a systematic review and meta-analysis
Source: JSES Rev Rep Tech. 2026 Sep 4;7(1):100875. doi: 10.1016/j.xrrt.2026.100875 (PMC13755963; doi:10.1016/j.xrrt.2026.100875)
Supplement: Supplementary file 4 [file mmc4.docx]

| **PICO Component** | **Inclusion Criteria** | **Exclusion Criteria** |
| --- | --- | --- |
| **Population (P)** | • Adult patients (≥18 years) undergoing primary total shoulder arthroplasty (TSA)  • All surgical indications (e.g. osteoarthritis, rotator cuff arthropathy, fracture, inflammatory arthritis)  • Population-based, registry, database, or cohort studies | • Paediatric patients (<18 years)  • Revision shoulder arthroplasty  • Non-arthroplasty shoulder procedures  • Cadaveric, biomechanical, or animal studies |
| **Intervention (I)** | • Reverse total shoulder arthroplasty (rTSA) performed as a primary procedure | • Revision rTSA  • Hemiarthroplasty or partial shoulder arthroplasty  • Hybrid or non-standard implant designs |
| **Comparison (C)** | • Anatomic total shoulder arthroplasty (aTSA)  • Studies directly comparing rTSA with aTSA  • Single-arm studies reporting outcomes for TSA, aTSA, or rTSA | • Studies without clear specification of arthroplasty type  • Comparisons involving hemiarthroplasty only  • Studies comparing TSA to non-operative management |
| **Outcomes (O)** | • Reported serious adverse events (SAEs)  • 30-day readmission rate  • 90-day readmission rate | • Studies reporting only long-term outcomes (>90 days)  • Functional scores only (e.g. ASES, Constant, ROM) without complication or readmission data  • Outcomes not clearly defined or not extractable |
| **Study Design / Setting** | • Observational studies (registry-based, administrative database, cohort studies)  • Full-text articles published in English | • Case reports, case series (<10 patients)  • Conference abstracts, editorials, letters, reviews  • Non-English publications |

Supplementary Table 2 – Inclusion and Exclusion criteria Figures
