# Supplementary material for: Serious adverse events and 30-day and 90-day re-admission rates following anatomic versus reverse total shoulder arthroplasty: a systematic review and meta-analysis
Source: JSES Rev Rep Tech. 2026 Sep 4;7(1):100875. doi: 10.1016/j.xrrt.2026.100875 (PMC13755963; doi:10.1016/j.xrrt.2026.100875)
Supplement: Supplementary file 5 [file mmc5.docx]

Supplementary Figure 3 - Funnel Plot aTSA vs rTSA 90-day Readmission
